# Vaccination Coverage Against Coronavirus Disease 2019 (COVID‐19) in Brazil's Indigenous Population

**DOI:** 10.1002/jmv.70965

**Published:** 2026-05-18

**Authors:** Nathália Mariana Santos Sansone, Patrícia Teixeira Costa, Lucas Silva Mello, Luiz Felipe Azevedo Marques, Vinícius Santiago dos Santos, Fernando Augusto Lima Marson

**Affiliations:** ^1^ Laboratory of Molecular Biology and Genetics, Postgraduate Program in Health Sciences, Postgraduate Program in Health Data Science São Francisco University (USF; acronym for *Universidade São Francisco*) Bragança Paulista São Paulo Brazil; ^2^ Laboratory of Clinical and Molecular Microbiology, Postgraduate Program in Health Sciences, Postgraduate Program in Health Data Science São Francisco University (USF; acronym for *Universidade São Francisco*) Bragança Paulista São Paulo Brazil; ^3^ LunGuardian Research Group – Epidemiology of Respiratory and Infectious Diseases, Postgraduate Program in Health Sciences Postgraduate Program in Health Data Science Bragança Paulista São Paulo Brazil

**Keywords:** epidemiology, Indigenous, pandemic, public health, SARS‐CoV‐2, vaccines, vulnerable population

## Abstract

Indigenous populations in Brazil were prioritized for coronavirus disease 2019 (COVID‐19) vaccination due to their increased vulnerability. The aim of the study was to describe COVID‐19 vaccination coverage among Indigenous population across Brazilian regions and Special Indigenous Health Districts (DSEIs; acronym for *Distritos Sanitários Especiais Indígenas*). A retrospective study was conducted using data from January 17, 2021, to June 27, 2024, obtained from the open‐access COVID‐19 vaccination database made available by the Brazilian Ministry of Health, covering the 4‐year period of the pandemic. The dataset included information on the Indigenous population (number of inhabitants), as well as the number and type of vaccine doses administered (first dose, second dose, single dose, and booster dose). Second dose refers to vaccines requiring a two‐dose primary schedule, whereas single‐dose refers to vaccines administered as a single primary dose; booster dose refers to any additional dose given after completion of the primary vaccination schedule and, according to the database, was restricted to individuals aged ≥ 18 years. Vaccination coverage was calculated by dividing the number of doses administered by the total Indigenous population, based on estimated from the 2022 Brazilian Institute of Geography and Statistics (IBGE; acronym for *Instituto Brasileiro de Geografia e Estatística*) Census, and multiplying by 100. The data was analyzed and presented according to Brazil's federative units (states and the Federal District) and DSEIs. A total of 1 490 036 COVID‐19 vaccine doses were administered to the Indigenous population. Of these, 631 990 (42%) corresponded to first doses, 551 602 (27%) to second or single doses, and 306 444 (21%) to booster doses. These doses corresponded to vaccination coverage of 89%, 78%, and 43% of the Indigenous population (*N* = 707 739), respectively. Across the 27 federative units, the lowest coverage for the first dose was observed in Tocantins (83%), Roraima (74%), and Pará (73%), while for second or single doses the lowest rates were found in Amapá (63%), Roraima (60%), and Pará (58%). Among the 34 DSEIs, 13 (38.2%) did not reach 90% coverage for the first dose, and 21 (61.8%) did not reach this threshold for the second or single doses. The lowest coverage levels were identified in Araguaia (65% and 44%), Rio Tapajós (59% and 40%), and Kaiapó do Pará (56% and 34%) for first and second or single doses, respectively. Vaccination coverage against COVID‐19 among Brazil's Indigenous population has not fully reached the 90% target recommended by the Brazilian government in alignment with the World Health Organization after 4 years of the pandemic. This gap may be explained by multiple factors, including cultural barriers, remote geographic conditions, and logistical challenges related to vaccine distribution.

## 1 Introduction

Brazil has an Indigenous population of approximately 1 693 535 people, comprising 305 ethnic groups and speaking 274 distinct languages [1, 2]. More than half (51.2%) of this population is concentrated in the Legal Amazon [3]. In recognition of their distinct sociocultural characteristics, Brazil's Unified Health System established the Special Indigenous Health Districts (DSEIs; acronym for *Distritos Sanitários Especiais Indígenas*), which are responsible for delivering primary healthcare services within Indigenous territories [4].

The Indigenous peoples of Brazil face inadequate health determinants, including limited access to safe drinking water and sanitation, barriers to prenatal care, and high rates of child malnutrition [5]. In addition, they face a high incidence of infectious and respiratory diseases [6, 7], like observed in the recent coronavirus disease 2019 (COVID‐19) pandemic in 2020 and influenza A virus subtype H1N1 in 2009 with high mortality in this population group [8, 9, 10, 11, 12].

Recently, infant mortality has been reported to be 60% higher among Indigenous peoples compared to the general population [5]. In addition, the health crisis affecting Indigenous communities has received significant media attention, highlighting issues such as the nutritional emergency and the resurgence of malaria among the Yanomami people in the states of Roraima and Amazonas [13]. Furthermore, outbreaks of sexually transmitted infections have been reported in the Xingu region, contributing to increased morbidity and mortality, including deaths from cervical cancer [14]. Given their distinct social and biological characteristics, Indigenous populations may face increased vulnerability to infectious diseases, which can be associated with disparities in immune response, as well as higher disease severity and mortality rates [15, 16].

Vaccination is one of the greatest achievements in public health, and Brazil has historically been a global reference in national immunization programs. However, in recent years, vaccination coverage rates have declined across the country [17, 18, 19, 20]. Since 2015, a marked reduction has been observed, with coverage for vaccines administered to children and adolescents falling below national targets [21]. A decade later, a partial recovery has emerged. Between 2024 and 2025, coverage increased for key vaccines, including the Pneumococcal 10‐valent Conjugate Vaccine (booster dose), from 80.66% to 89.13%, and the Bacillus Calmette–Guérin (BCG) vaccine, from 63.59% to 88.29% [22]. Despite these improvements, coverage levels remain below the national target of 95%.

These figures highlight the persistence of suboptimal vaccination coverage in Brazil, even in the context of recent gains. This gap is even more pronounced among vulnerable populations, particularly Indigenous peoples, who face limited access to health services and structural barriers to healthcare delivery [23, 24]. A similar pattern was observed during the COVID‐19 pandemic, when vaccination coverage among Indigenous populations remained lower than that of the general Brazilian population [25], contributing to disproportionately high mortality rates associated with severe acute respiratory syndrome coronavirus 2 (SARS‐CoV‐2) infection [2, 26, 27]. Taken together, these findings underscore that a substantial proportion of preventable morbidity and mortality remains inadequately addressed. Strengthening vaccination strategies and expanding equitable access to healthcare are essential to mitigate avoidable diseases and improve health outcomes, particularly in historically underserved populations [28].

In this regard, studies have shown heterogeneity in the process of COVID‐19 immunization among Indigenous communities [29, 30, 31]. In March 2021, some DSEIs achieved high vaccination coverage, while others remained below 60% coverage until December 2021, particularly in the North and Central‐West regions of Brazil [24]. This scenario reflects the inherent challenges of vaccinating more isolated communities. However, during the H1N1 pandemic, 90% of the Indigenous population was vaccinated within a 3‐month period, indicating that geographical isolation alone cannot explain the vaccination coverage observed for COVID‐19 [24].

The vaccine hesitancy movement in this context is multifactorial and has been increasingly shaped by the interaction between political ideologies, institutional trust, and the rapid dissemination of misinformation, particularly through digital media ecosystems [32, 33]. In Brazil, the politicization of the COVID‐19 pandemic contributed to conflicting public health messages, which weakened risk perception and reduced confidence in vaccination campaigns. This dynamic was further amplified by the widespread circulation of fake news on social media platforms and messaging applications, often disseminating misleading claims about vaccine safety, efficacy, and potential adverse effects [9, 34]. In Indigenous contexts, where access to reliable, culturally adapted information may be limited, such misinformation can have a disproportionate impact, fostering uncertainty and fear. Moreover, historical processes of marginalization and previous negative experiences with governmental institutions may intensify skepticism toward externally driven health interventions. Added to this, distrust in public institutions and in the scientific community, frequently reinforced by inconsistent communication strategies and lack of culturally sensitive engagement, further contributes to lower adherence to COVID‐19 vaccination [35]. Together, these factors highlight that vaccine hesitancy is not merely an individual choice, but a complex social phenomenon influenced by political, informational, and structural determinants.

Given the scarcity of epidemiological data on Indigenous peoples, this study aimed to describe COVID‐19 vaccination coverage in this population in Brazil over 4 years of the pandemic, compare it with the target established by the World Health Organization (WHO), and explore factors associated with low uptake, such as geographical barriers, infrastructure limitations, trust in institutions, and the spread of misinformation. The results of this study can guide the strengthening of Indigenous health policies by directly informing the design and prioritization of vaccination strategies tailored to the specific realities of each DSEI, particularly by highlighting regional disparities that require a more equitable and decentralized allocation of resources. By identifying operational and contextual barriers to vaccination, the findings provide actionable evidence to support targeted interventions, such as micro‐planning strategies for active case finding in hard‐to‐reach areas and the optimization of logistics for vaccine delivery. Furthermore, the results reinforce the importance of culturally adapted, intercultural communication strategies aligned with the sociocultural characteristics of each ethnic group to reduce vaccine hesitancy and improve community engagement. Finally, the analysis of data gaps offers a technical basis for strengthening and integrating health information systems, enabling more accurate monitoring of vaccination coverage and more timely responses to future public health emergencies in Indigenous territories.

## 2 Methods

This is a cross‐sectional epidemiological study conducted from January 17, 2021, to June 27, 2024, using the open‐access database on COVID‐19 vaccination coverage provided by the Brazilian Ministry of Health [36]. The platform was developed by the Department of Monitoring, Evaluation and Dissemination of Strategic Health Information of the Secretariat of Information and Digital Health, in partnership with the Department of Immunization and Vaccine‐preventable Diseases of the Secretariat of Health and Environmental Surveillance [36].

The study period encompasses distinct phases of the COVID‐19 vaccination campaign in Brazil, including the initial rollout of monovalent vaccines, the administration of booster doses, and the subsequent introduction of bivalent vaccines. Given this dynamic transition in vaccination strategies, the present study was designed to provide a cross‐sectional assessment of vaccination coverage at a stage characterized by epidemiological stabilization and pandemic control, rather than a temporal trend analysis.

### 2.1 Data Source and Extraction

COVID‐19 vaccination data are updated daily and originate exclusively from the National Health Data Network. The platform provides multiple filtering options, including geographic (region, federative unit, and municipality), temporal (year, month, and date of vaccination), and individual and vaccination‐related variables (vaccine type, number of doses, and sex). For this study, only a subset of these variables was selected according to the analytical objectives.

In this context, data related to COVID‐19 vaccination among Indigenous individuals served by Indigenous Health Subsystem of the Brazilian Unified Health System (SASI‐SUS; acronym for *Subsistema de Atençã*o à *Saúde Indígena do Sistema Único de Saúde*) were extracted and analyzed. The dataset was structured into two components: aggregated (total) data and stratified (fractional) data. Aggregated data included the total Indigenous population (number of registered individuals) and the overall number and type of vaccine doses administered (first dose, second or single dose, and booster dose, the latter restricted to individuals aged ≥ 18 years). Stratified data described the distribution of vaccinated Indigenous individuals across Brazil, according to DSEIs and the corresponding federative units in which each DSEI is administratively based.

Second dose refers to vaccines requiring a two‐dose primary schedule, whereas single‐dose refers to vaccines administered as a single primary dose; booster dose refers to any additional dose given after completion of the primary vaccination schedule.

### 2.2 Study Variables and Definitions

Age was categorized into three groups (3–4 years, 5–17 years, and ≥ 18 years); booster doses were reported exclusively for individuals aged ≥ 18 years, according to the database.

Vaccination coverage was calculated as the ratio between the number of administered doses and the Indigenous population, multiplying by 100. For the purposes of this study, the Indigenous population was defined according to the Brazilian Institute of Geography and Statistics (IBGE; acronym for *Instituto Brasileiro de Geografia e Estatística*) as individuals who self‐identified as Indigenous based on color or race, or self‐recognition, regardless of residing within or outside Indigenous territories.

It is important to note that the population assigned to each DSEI corresponds to the Indigenous population registered in the Indigenous Health Care Information System (SIASI; acronym for *Sistema de Informação da Atenção à Saúde Indígena*), reflecting an ascribed population defined by ethno‐cultural and territorial criteria. This includes individuals living within officially recognized Indigenous Lands as well as those residing in nearby communities linked to these territories. Therefore, the concept of “resident” within a DSEI is not strictly limited to geographically demarcated areas but rather encompasses the broader Indigenous population under the responsibility of each district.

Given the use of aggregated data and the organizational structure of DSEIs, it was not possible to assess or control for population mobility (e.g., movement on and off Indigenous lands during the pandemic). Such dynamics are not captured at the level of detail available in the database and, therefore, could not be explicitly incorporated into the analyses.

### 2.3 Outcome Measures and Analysis

In the study, a vaccination coverage threshold of 90% was adopted as a parameter of vaccination success. This benchmark was used for comparative purposes for first dose and second or single‐dose coverage; however, it is primarily applicable in epidemiological contexts involving complete vaccination schedules, such as two‐dose monovalent regimens (second dose) or single‐dose vaccines. The threshold is consistent with targets established by the Brazilian Ministry of Health, in alignment with recommendations from the WHO [37, 38].

The 2022 IBGE Census of the Brazilian Institute of Geography and Statistics, Brazil's official data and statistics agency, was used to obtain the number of Indigenous inhabitants per DSEI. In addition, supporting data were collected from the Brazilian Ministry of Health's Special Secretariat for Indigenous Health (SESAI; acronym for Secretaria Especial de Saúde Indígena) regarding the number of base centers, villages, ethnic groups, Basic Indigenous Health Units (UBSIs; acronym for *Unidades Básicas de Saúde Indígena*), and Indigenous Health Houses (CASAIs; acronym for *Casas de Saúde Indígena*) within each DSEI. Data on monovalent and bivalent vaccination coverage for the total population of Brazil, according to federative units (states and the Federal District), was also collected.

For monovalent vaccination coverage, the following indicators were obtained: (a) number of doses administered (first dose, second dose, third dose, and single dose), (b) number of individuals with complete vaccination (two doses of monovalent vaccines or one dose of single‐dose vaccine), (c) number of booster doses administered (first booster dose, second booster dose, third booster dose, and additional doses), (d) total number of doses administered, and (e) total population.

For bivalent vaccination coverage, the following indicators were collected: (a) number of doses administered according to vaccination strategy (booster dose and other booster doses), (b) total number of bivalent doses administered, and (c) total population.

Vaccination coverage was calculated using the formula described aboveː [number of doses/Indigenous population] × 100.

The number of vaccine doses administered per inhabitant was estimated as the ratio between the total number of doses administered (monovalent and bivalent) and the total population of each federative unit. This metric reflects the intensity of vaccine delivery relative to population size.

For comparative analyses involving the general Brazilian population and Indigenous peoples, complete vaccination coverage was defined as the proportion of individuals who received either at least two doses of monovalent vaccines or one dose of a single‐dose vaccine. This combined metric reflects updated national immunization guidelines and allows a more comprehensive estimation of effective immunization coverage in the context of evolving vaccination strategies.

The analyses were conducted using aggregated secondary data obtained from a national open‐access platform maintained by the Brazilian Ministry of Health, which represents the primary official source of epidemiological data in Brazil. Due to the absence of access to individual‐level records, it was not possible to assess data completeness, identify missing values, or apply statistical methods to manage incomplete information (e.g., imputation or sensitivity analyses).

Although these limitations are inherent to the use of aggregated secondary data, the dataset covers a large population base at the national level, which contributes to the robustness of the estimates. Nonetheless, the study remains subject to potential underreporting and inconsistencies in the recording of Indigenous status, which should be considered when interpreting the findings.

### 2.4 Organization of Indigenous Healthcare in Brazil

To support the interpretation of vaccination coverage findings, a brief contextual description of the organization of Indigenous healthcare in Brazil is presented below. This section is not part of the analytical dataset but is included to provide structural and operational context for understanding the distribution, access, and delivery of vaccination services among Indigenous populations. The information described herein serves solely as a framework to facilitate the interpretation of results and discussion, without being incorporated into the statistical analysis.

The Brazilian Indigenous healthcare system is organized through a differentiated and culturally adapted model of care, designed to address the specific sociocultural, geographic, and epidemiological characteristics of Indigenous populations. This model is implemented through the DSEIs, which function as decentralized administrative units responsible for coordinating healthcare delivery within defined territories.

DSEIs operate under the SESAI and are structured based on epidemiological, geographic, and ethnographic criteria. Healthcare provision follows a primary care‐oriented approach, centered on UBSIs located within Indigenous lands. These units are supported by multidisciplinary teams that include both Indigenous health agents and non‐Indigenous healthcare professionals.

To ensure access in remote areas, the system incorporates itinerant healthcare teams that periodically travel to Indigenous communities, overcoming geographic barriers. For cases requiring specialized care, patients are referred to CASAIs located in urban centers, where they and their families can stay during treatment. This structure enables access to secondary and tertiary care services that are not available within Indigenous territories.

This integrated framework aims to promote accessibility, continuity of care, and cultural appropriateness. However, its effectiveness depends on factors such as infrastructure, logistics, and communication, which are critical determinants of health outcomes, including vaccination coverage.

### 2.5 Data Visualization and Statistical Software

The data were organized into tables and illustrative figures. The figures presented in the article were created in GraphPad Prism version 10.2.3 for Mac (GraphPad Software, Boston, MA, USA; www.graphpad.com).

## 3 Results

### 3.1 Overall COVID‐19 Vaccination Coverage According to Dose Type Among Indigenous Populations in Brazil

**Figure 1:**
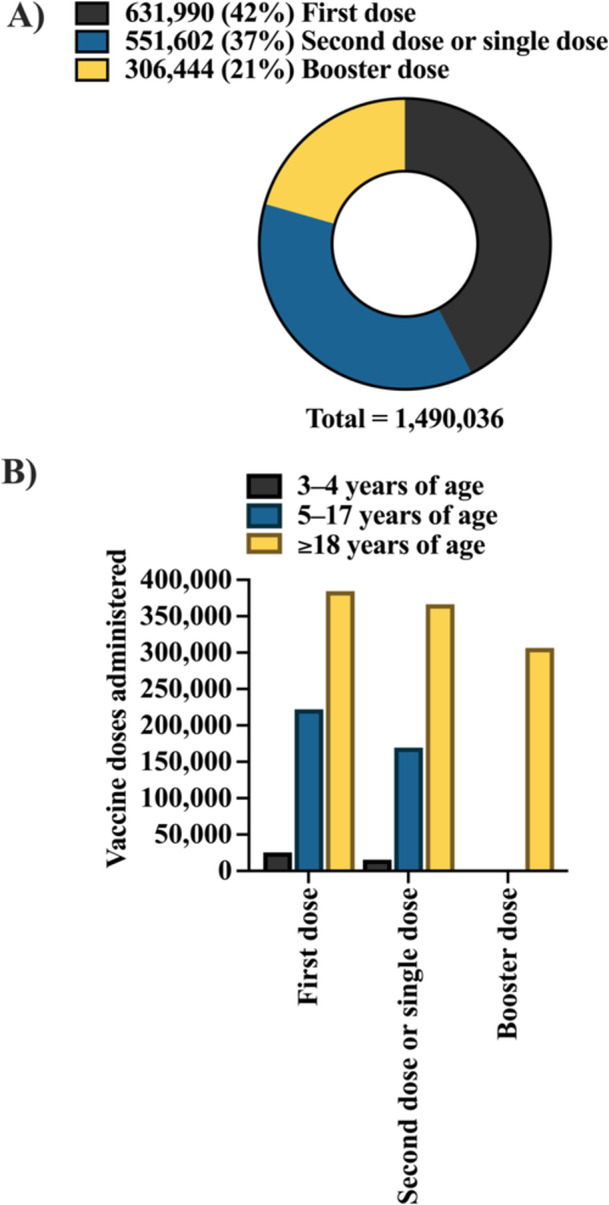
Total number of coronavirus disease 2019 (COVID‐19) vaccine doses administered to the Indigenous population in Brazil, stratified by dose type (A) and age group (B). Panel (A) presents the distribution of administered doses according to vaccine dose category (e.g., first dose, second dose, booster dose), allowing the visualization of the progression of vaccination strategies. Panel (B) shows the distribution by age group, highlighting differences in vaccine uptake across the lifespan. Data are presented as absolute numbers (*N*) and percentage (%), enabling both quantitative comparison and proportional interpretation. Data were obtained from the National Health Data Network (OpenDataSUS), Ministry of Health, Federal Government of Brazil. The data correspond to the period from January 17, 2021, to June 27, 2024. The complete dataset is available at: https://www.gov.br/saude/pt‐br/.

A total of 1 490 036 doses of COVID‐19 vaccines were administered to Brazil's Indigenous population. Of this amount, the majority was related to first doses [*N* = 631 990 (42%)], followed by second or single doses [*N* = 551 602 (37%)] and booster doses, restricted to individuals aged ≥ 18 years [*N* = 306 444 (21%)]. These corresponded to vaccination coverage of 89%, 78%, and 43% of the Indigenous population (*N* = 707 739), respectively (Table 1, Figure 1a).

**Table 1:** Total number of coronavirus disease 2019 (COVID‐19) vaccine doses administered to the Indigenous population of Brazil, by dose type.

Category	Total (%)^b^^,^ c
Indigenous population	707 739
Total doses administered	1 490 036
First dose	631 990 (89%)
Second dose or single dose	551 602 (78%)
Booster dose^a^	306 444 (43%)

*Note:* Data were obtained from the National Health Data Network (OpenDataSUS), Ministry of Health, Federal Government of Brazil. The data correspond to the period from January 17, 2021, to June 27, 2024. The complete dataset is available at: https://www.gov.br/saude/pt‐br/. Data are presented as absolute numbers (*N*) and percentage (%), enabling both quantitative comparison and proportional interpretation.

^a^ Available only for Indigenous individuals over ≥ 18 years.

^b^ Percentage represent vaccination coverage based on the total Indigenous population. Vaccination coverage was calculated using the formula (number of administered doses/Indigenous population) × 100.

^c^ In the studied population, first doses, second or single doses, and booster doses accounted for 42%, 37%, and 21% of the total doses administered, respectively.

It is important to highlight that these findings reflect a consolidated stage of the national vaccination campaign, following a dynamic transition in vaccination strategies from primary monovalent schemes to booster and bivalent doses, corresponding to a period of epidemiological stabilization of COVID‐19 in Brazil.

### 3.2 Age‐Stratified Distribution of COVID‐19 Vaccine Doses Across Indigenous Populations and DSEIs

Regarding the total number of vaccine doses administered by age group, the highest number was observed among individuals aged ≥ 18 years (*N* = 1 057 272), followed by those aged 5–17‐years (N = 391 626) and 3–4 years (*N* = 41 138) (Figure 1b).

In all three age groups, the number of first doses exceeded the number of second or single doses, with the greatest difference observed in the 5–17 age group, where first doses were 76% higher than second or single doses (Table 2, Figure 1b).

**Table 2:** Distribution of coronavirus disease 2019 (COVID‐19) vaccine doses by age group and by Special Indigenous Health Districts (DSEIs; acronym for *Distritos Sanitários Especiais Indígenas*).^a^

DSEIs	First dose 1^b^			Second dose or single dose^b^			Booster dose^a^^,^ b
	3–4 years	5–17 years	≥ 18 years	3–4 years	5–17 years	≥ 18 years	≥ 18 years
Alagoas and Sergipe	481 (4%)	3615 (29%)	8531 (67%)	426 (3%)	3445 (28%)	8412 (69%)	7714 (100%)
Altamira	271 (6%)	1822 (43%)	2193 (51%)	176 (4%)	1692 (42%)	2179 (54%)	2209 (100%)
Alto Rio Juruá	692 (5%)	5086 (40%)	7121 (55%)	482 (5%)	3245 (33%)	6232 (62%)	4988 (100%)
Alto Rio Negro	1706 (7%)	7003 (28%)	16 080 (65%)	688 (3%)	7003 (29%)	16 080 (68%)	16 080 (100%)
Alto Rio Purus	639 (6%)	4021 (40%)	5497 (54%)	193 (2%)	2541 (33%)	5039 (65%)	3868 (100%)
Alto Rio Solimões	1596 (3%)	23 332 (43%)	29 865 (54%)	452 (1%)	11 059 (29%)	26 167 (70%)	19 007 (100%)
Amapá and Norte do Pará	383 (4%)	3743 (38%)	5644 (58%)	111 (2%)	1906 (26%)	5099 (72%)	4840 (100%)
Araguaia	0 (0%)	1186 (36%)	2065 (64%)	0 (0%)	379 (17%)	1834 (83%)	1164 (100%)
Bahia	1039 (3%)	8908 (29%)	20 764 (68%)	785 (3%)	7985 (27%)	20 488 (70%)	18 660 (100%)
Ceará	986 (4%)	6322 (23%)	20 312 (73%)	986 (4%)	6322 (23%)	20 224 (73%)	19 040 (100%)
Cuiabá	236 (3%)	2973 (37%)	4787 (60%)	217 (3%)	2382 (33%)	4697 (64%)	4910 (100%)
Guamá‐Tocantins	611 (4%)	5702 (36%)	9619 (60%)	94 (1%)	4366 (33%)	8845 (66%)	8259 (100%)
Interior Sul	1480 (4%)	5702 (30%)	21 637 (66%)	1139 (4%)	8329 (27%)	21 354 (69%)	17 796 (100%)
Kaiapó do Mato Grosso	264 (6%)	9776 (44%)	2229 (50%)	178 (5%)	1523 (46%)	1633 (49%)	1613 (100%)
Kaiapó do Pará	76 (2%)	1969 (32%)	2133 (66%)	12 (1%)	192 (10%)	1781 (90%)	749 (100%)
Leste de Roraima	324 (1%)	12 921 (35%)	23 469 (64%)	65 (0%)	9215 (30%)	21 490 (70%)	12 829 (100%)
Litoral Sul	1066 (5%)	7918 (34%)	13 888 (61%)	729 (3%)	7631 (35%)	13 632 (62%)	13 031 (100%)
Manaus	1367 (5%)	10 803 (38%)	16 146 (57%)	1060 (4%)	9616 (36%)	15 876 (60%)	14 549 (100%)
Maranhão	2184 (6%)	12 952 (39%)	18 465 (55%)	1076 (4%)	10 766 (36%)	17 795 (60%)	14 679 (100%)
Mato Grosso do Sul	1962 (3%)	24 348 (36%)	41 441 (61%)	1071 (2%)	16 981 (29%)	40 022 (69%)	31 495 (100%)
Médio Rio Purus	409 (6%)	2294 (34%)	3971 (60%)	214 (4%)	1741 (31%)	3648 (65%)	3072 (100%)
Médio Rio Solimões and Afluentes	0 (0%)	5799 (40%)	8520 (60%)	0 (0%)	1616 (16%)	8406 (84%)	6439 (100%)
Minas Gerais and Espírito Santo	641 (4%)	5431 (33%)	10 517 (63%)	560 (4%)	4934 (31%)	10 285 (65%)	9031 (100%)
Parintins	960 (7%)	5203 (40%)	6847 (53%)	960 (8%)	5038 (40%)	6555 (52%)	6168 (100%)
Pernambuco	1609 (4%)	10 523 (28%)	25 644 (68%)	1536 (4%)	10 523 (28%)	25 547 (68%)	24 224 (100%)
Porto Velho	452 (5%)	3817 (39%)	5590 (57%)	288 (3%)	3501 (38%)	5481 (59%)	5526 (100%)
Potiguara	727 (5%)	4240 (29%)	9488 (66%)	566 (4%)	4070 (29%)	9349 (67%)	8714 (100%)
Rio Tapajós	128 (2%)	2306 (29%)	5363 (69%)	44 (1%)	847 (16%)	4372 (83%)	1456 (100%)
Tocantins	129 (1%)	4213 (42%)	5628 (57%)	0 (0%)	2519 (33%)	5122 (67%)	3223 (100%)
Vale do Javari	281 (6%)	1980 (39%)	2798 (55%)	129 (3%)	1373 (33%)	2716 (64%)	1986 (100%)
Vilhena	233 (4%)	2207 (39%)	3172 (57%)	153 (3%)	2025 (38%)	3104 (59%)	2969 (100%)
Xavante	1252 (7%)	8334 (44%)	9458 (49%)	769 (4%)	7859 (44%)	9229 (52%)	8161 (100%)
Xingu	394 (6%)	2516 (40%)	3369 (54%)	164 (3%)	2321 (39%)	3455 (58%)	3204 (100%)
Yanomami	903 (4%)	7843 (38%)	12 076 (58%)	334 (2%)	4529 (30%)	10 323 (68%)	4791 (100%)

*Note:* Data were obtained from the National Health Data Network (OpenDataSUS), Ministry of Health, Federal Government of Brazil. The data correspond to the period from January 17, 2021, to June 27, 2024. The complete dataset is available at: https://www.gov.br/saude/pt‐br/. Data are presented as absolute numbers (*N*) and percentage (%), enabling both quantitative comparison and proportional interpretation.

^a^ Available only for Indigenous individuals over ≥ 18 years.

^b^ Percentage represent vaccination coverage based on the total Indigenous population. Vaccination coverage was calculated using the formula (number of administered doses/Indigenous population) × 100.

Examining the distribution of doses by age group across DSEIs, for the first dose, coverage in the 3–4 year age group ranged from 0% (Araguaia and Médio Rio Solimões and Afluentes) to 7% (Alto Rio Negro, Parintins, and Xavante). Among individuals aged 5–17 years, the range was 23% (Ceará) and 44% (Kaiapó do Pará and Xavante). For those aged ≥ 18 years, the lowest percentage of first doses was recorded in Kaiapó do Pará and Xavante (49%), while the highest was observed in Ceará (73%) (Table 2, Figure 1b).

For second or single doses, in the 3–4‐year age group, three DSEIs reported no data (Araguaia, Médio Rio Solimões and Afluentes, and Tocantins), while the highest value was observed in Parintins (8%). In the 5–17 age group, Kaiapó do Pará had the lowest proportion (10%), whereas Kaiapó do Mato Grosso had the highest (46%). Among individuals aged ≥ 18 years, Kaiapó do Pará had the highest coverage (90%), while Kaiapó do Mato Grosso had the lowest (49%). Booster doses were administered exclusively to individuals aged ≥ 18 years (Table 2, Figure 1b).

### 3.3 Geographic Variation in Vaccination Coverage Across Brazil's Federative Units

**Figure 2:**
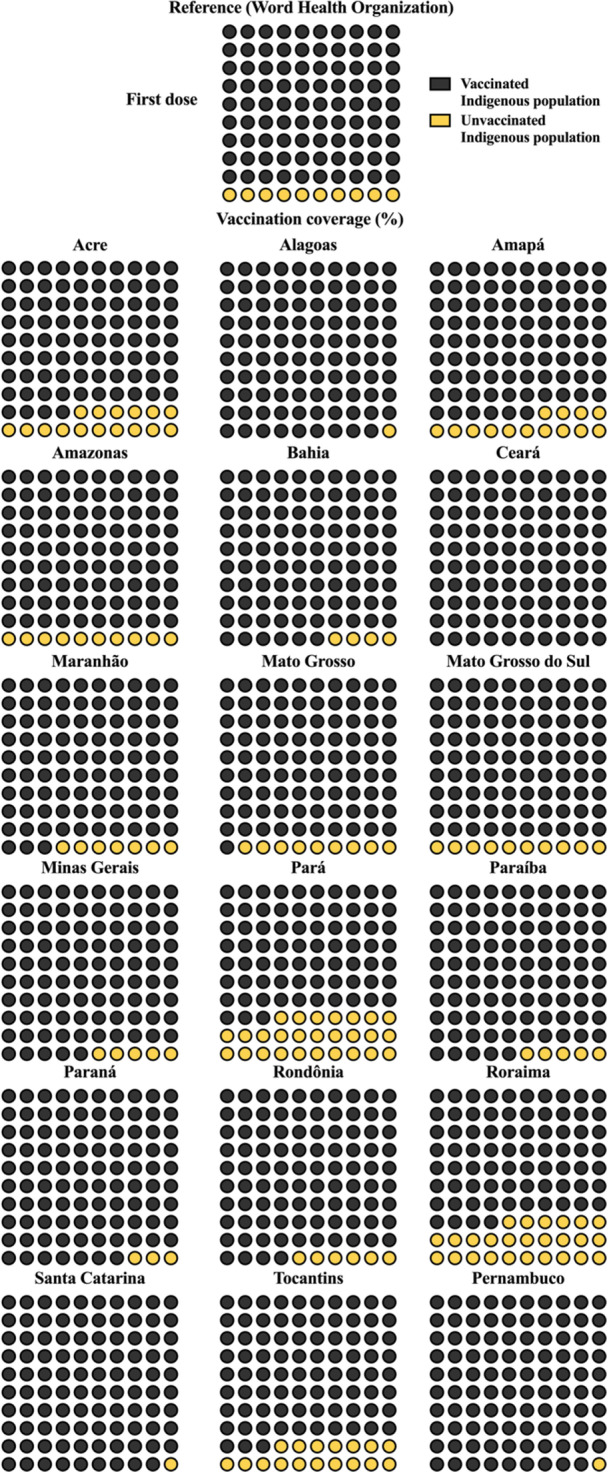
Vaccination coverage for the first dose of coronavirus disease 2019 (COVID‐19) vaccine among Indigenous population in Brazil, stratified by Special Indigenous Health Districts (DSEIs; acronym for *Distritos Sanitários Especiais Indígenas*). Each panel represents a specific DSEI and is displayed as a 10 × 10 unit chart (waffle chart), where each of the 100 cells corresponds to 1% of the total Indigenous population in that region. This structure allows direct visual estimation of vaccination coverage without relying on abstract proportions. Dark‐colored cells represent individuals who received the first vaccine dose (vaccinated population), whereas light‐colored cells indicate individuals who have not yet been vaccinated (coverage gap). This contrast enables immediate identification of both achieved coverage and remaining deficits. At the top of the figure, the “Reference” panel represents the 90% vaccination coverage target recommended by the World Health Organization (WHO), serving as a visual benchmark for comparison. By comparing each DSEI panel to this reference, readers can quickly identify regions that have reached, approached, or remain below the recommended threshold. The data is presented in percentage (%). Vaccination coverage was calculated using the formula (number of administered doses/Indigenous population) × 100. Data were obtained from the National Health Data Network (OpenDataSUS), Ministry of Health, Federal Government of Brazil. The data correspond to the period from January 17, 2021, to June 27, 2024. The complete dataset is available at: https://www.gov.br/saude/pt‐br/.

Of Brazil's 27 federative units, 18 include DSEIs. For first‐dose coverage, only Ceará reached 100%, while Pernambuco, Santa Catarina, and Alagoas reached 99%. Among the remaining states, nine had coverage between 90% and 97%, with the lowest values observed in Tocantins (83%), Roraima (74%), and Pará (73%) (Table 3, Figure 2).

**Table 3:** Percentage distribution of coronavirus disease 2019 (COVID‐19) vaccine doses among Indigenous population in Brazil, by federative unit of the headquarters of the Special Indigenous Health Districts (DSEIs; acronym for *Distritos Sanitários Especiais Indígenas*)^a^.

Federative unit with DSEI headquarters	First dose	Second dose or single dose
Acre	84%	65%
Alagoas	99%	96%
Amapá	86%	63%
Amazonas	90%	74%
Bahia	96%	92%
Ceará	100%	99%
Maranhão	93%	82%
Mato Grosso	91%	81%
Mato Grosso do Sul	90%	77%
Minas Gerais	95%	90%
Pará	73%	58%
Paraíba	95%	91%
Paraná	97%	93%
Rondônia	94%	89%
Roraima	74%	60%
Santa Catarina	99%	92%
Tocantins	83%	64%
Pernambuco	99%	99%

*Note:* Data were obtained from the National Health Data Network (OpenDataSUS), Ministry of Health, Federal Government of Brazil. The data correspond to the period from January 17, 2021, to June 27, 2024. The complete dataset is available at: https://www.gov.br/saude/pt‐br/. Data are presented as percentage (%).

^a^ Percentage represent vaccination coverage based on the total Indigenous population. Vaccination coverage was calculated using the formula (number of administered doses/Indigenous population) × 100.

**Figure 3:**
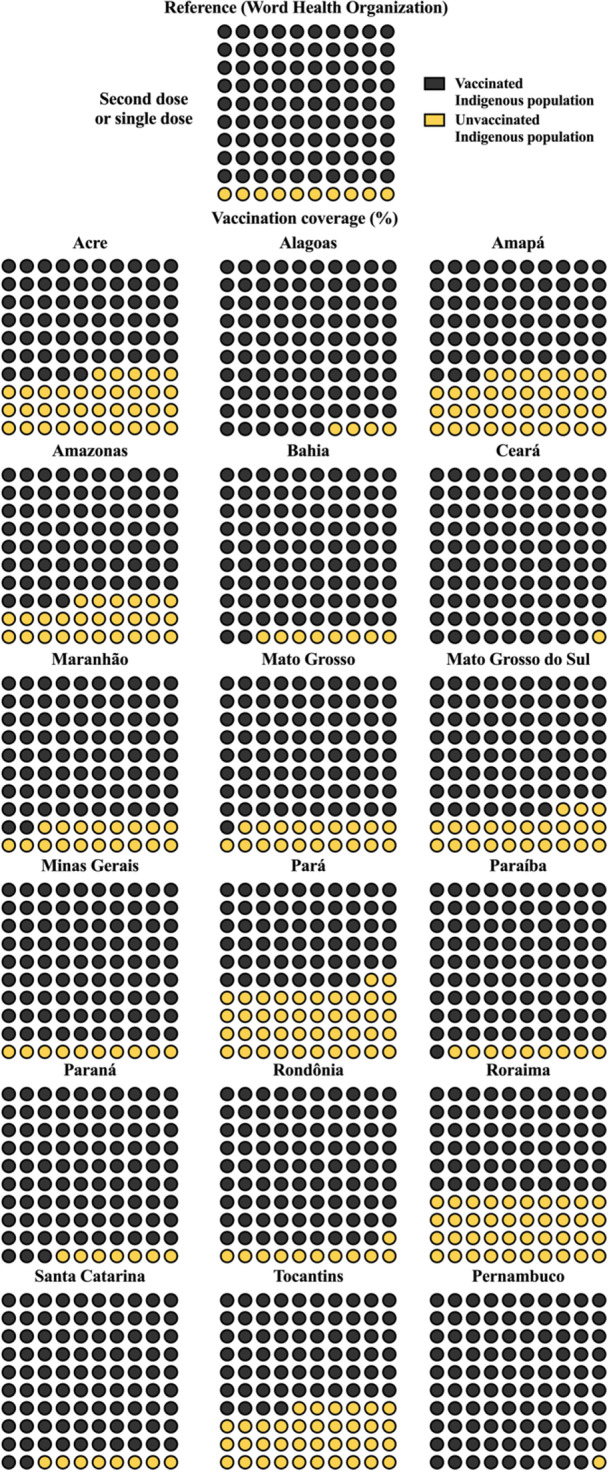
Vaccination coverage for complete coronavirus disease 2019 (COVID‐19) immunization (second dose or single‐dose regimen) among Indigenous population in Brazil, stratified by Special Indigenous Health Districts (DSEIs; acronym for *Distritos Sanitários Especiais Indígenas*). Each panel represents a specific DSEI and is displayed as a 10 × 10 unit chart (waffle chart), where each of the 100 cells corresponds to 1% of the total Indigenous population in that region. This structure allows direct visual estimation of vaccination coverage without relying on abstract proportions. Dark‐colored cells indicate individuals who completed the vaccination schedule (fully vaccinated), while light‐colored cells represent the proportion of the population that has not yet completed immunization. This distinction highlights not only overall coverage but also the magnitude of incomplete vaccination. At the top of the figure, the “Reference” panel represents the 90% vaccination coverage target recommended by the World Health Organization (WHO), serving as a visual benchmark for comparison. By comparing each DSEI panel to this reference, readers can quickly identify regions that have reached, approached, or remain below the recommended threshold. The data is presented in percentage (%). Vaccination coverage was calculated using the formula (number of administered doses/Indigenous population) × 100. Data were obtained from the National Health Data Network (OpenDataSUS), Ministry of Health, Federal Government of Brazil. The data correspond to the period from January 17, 2021, to June 27, 2024. The complete dataset is available at: https://www.gov.br/saude/pt‐br/.

For second or single doses, no federative unit reached 100% coverage. Ceará and Pernambuco achieved 99%, while only six states reached coverage between 90% to 100%, and five between 70% to 89% range. The lowest coverage was observed in Amapá (63%), Roraima (60%), and Pará (58%) (Table 3, Figure 3).

Considering the 90% target recommended by the WHO, 8 (44.4%) and 10 (55.6%) of federative units did not reach this threshold for first and second or single doses, respectively.

### 3.4 Heterogeneity of Vaccination Coverage Across DSEIs

**Figure 4:**
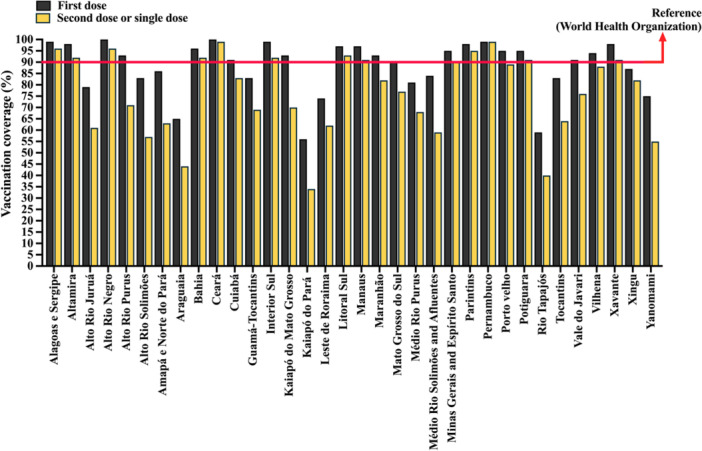
Overall coronavirus disease 2019 (COVID‐19) vaccination coverage among Indigenous populations in Brazil by Special Indigenous Health Districts (DSEIs; acronym for *Distritos Sanitários Especiais Indígenas*). This figure presents the aggregated vaccination coverage for each DSEI, allowing comparison of overall immunization performance across regions. Differences between districts reflect variations in access to healthcare services, logistics, and local vaccination strategies. Coverage is expressed as a percentage (%) and calculate as (number of administered doses/Indigenous population] × 100. Data were obtained from the National Health Data Network (OpenDataSUS), Ministry of Health, Federal Government of Brazil. The data correspond to the period from January 17, 2021, to June 27, 2024. The complete dataset is available at: https://www.gov.br/saude/pt‐br/.

A total of 34 DSEIs were evaluated. For first‐dose coverage, Ceará and Alto Rio Negro reached 100%, while Pernambuco and Interior Sul reached 99%. Among the remaining districts, 23 achieved coverage between 80% and 100%, with the lowest values observed in Araguaia (65%), Rio Tapajós (59%), and Kaiapó do Pará (56%) (Table 4, Figure 4).

**Table 4:** Profile of Brazil's Indigenous health management system and distribution of coronavirus disease vaccine 2019 (COVID‐19) vaccine doses in this population by Special Indigenous Health District (DSEIs; acronym for *Distritos Sanitários Especiais Indígenas*) and total population of each DSEI^a^^,^ b.

DSEIs	Population	First dose [*N* (%)]	Second dose or single dose [*N* (%)]	Total	Base centers	Villages	Ethnic groups	CASAI	UBSI
Alagoas and Sergipe	12 818	12 627 (99%)	12 283 (96%)	24 910	13	30	19	1	10
Altamira	4383	4286 (98%)	4047 (92%)	8333	1	129	21	1	31
Alto Rio Juruá	16 361	12 899 (79%)	9959 (61%)	22 858	7	145	32	1	1
Alto Rio Negro	24 789	24 789 (100%)	23 771 (96%)	48 560	25	677	48	1	5
Alto Rio Purus	10 968	10 157 (93%)	7773 (71%)	17 930	7	148	26	1	9
Alto Rio Solimões	65 706	54 793 (83%)	37 678 (57%)	92 471	12	220	34	1	15
Amapá and Norte do Pará	11 311	9770 (86%)	7116 (63%)	16 886	6	163	13	2	23
Araguaia	4992	3251 (65%)	2213 (44%)	5464	4	40	20	1	16
Bahia	31 927	30 741 (96%)	29 258 (92%)	59 999	9	130	27	1	24
Ceará	27 697	27 620 (100%)	27 532 (99%)	55 152	9	104	22	1	11
Cuiabá	8757	7996 (91%)	7296 (83%)	15 292	11	213	39	3	29
Guamá‐Tocantins	19 250	15 932 (83%)	13 305 (69%)	29 237	8	248	59	5	28
Interior Sul	33 359	32 893 (99%)	30 822 (92%)	63 715	12	198	22	0	70
Kaiapó do Mato Grosso	4778	4462 (93%)	3334 (70%)	7796	3	60	21	3	3
Kaiapó do Pará	5855	3255 (56%)	1985 (34%)	5240	4	77	5	4	18
Leste de Roraima	49 616	36 714 (74%)	30 770 (62%)	67 484	34	337	30	1	211
Litoral Sul	23 558	22 872 (97%)	21 992 (93%)	44 864	15	137	29	1	47
Manaus	29 124	28 316 (97%)	26 552 (91%)	54 868	17	266	54	1	4
Maranhão	36 017	33 601 (93%)	29 637 (82%)	63 238	6	640	28	3	44
Mato Grosso do Sul	75 542	67 751 (90%)	58 074 (77%)	125 825	14	99	40	3	79
Médio Rio Purus	8233	6674 (81%)	5603 (68%)	12 277	10	123	13	2	13
Médio Rio Solimões and Afluentes	17 040	14 319 (84%)	10 022 (59%)	24 341	15	189	25	2	18
Minas Gerais and Espírito Santo	17 515	16 589 (95%)	15 779 (90%)	32 368	26	98	29	2	8
Parintins	13 249	13 010 (98%)	12 553 (95%)	25 563	12	120	13	3	12
Pernambuco	38 139	37 776 (99%)	37 606 (99%)	75 382	12	218	21	1	59
Porto velho	10 415	9859 (95%)	9270 (89%)	19 129	5	197	61	6	27
Potiguara	15 292	14 455 (95%)	13 985 (91%)	28 440	3	35	8	0	22
Rio Tapajós	13 130	7797 (59%)	5263 (40%)	13 060	11	171	12	4	18
Tocantins	11 963	9970 (83%)	7641 (64%)	17 611	6	190	21	2	32
Vale do Javari	5563	5059 (91%)	4218 (76%)	9277	7	66	10	1	21
Vilhena	5996	5612 (94%)	5282 (88%)	10 894	4	167	41	4	48
Xavante	19 522	19 044 (98%)	17 857 (91%)	36 901	6	351	6	2	25
Xingu	7258	6279 (87%)	5940 (82%)	12 219	4	136	34	3	4
Yanomami	27 616	20 822 (75%)	15 186 (55%)	36 008	37	378	14	1	31

*Note:* Data are presented as absolute numbers (N) and percentage (%), representing vaccination coverage. Percentage represent vaccination coverage based on the total Indigenous population. Vaccination coverage was calculated using the formula (number of administered doses/Indigenous population) × 100.

^a^ Data were obtained from the National Health Data Network (OpenDataSUS), Ministry of Health, Federal Government of Brazil. The data correspond to the period from January 17, 2021, to June 27, 2024. The complete dataset is available at: https://www.gov.br/saude/pt‐br/

^b^ Data were obtained from the Special Secretariat for Indigenous Health, Ministry of Health, Federal Government of Brazil. These data refer to the number of base centers, villages, ethnic groups, Indigenous Health Houses (CASAI; acronym for *Casas de Saúde Indígena*), and Basic Indigenous Health Units (UBSI; acronym for *Unidades Básicas de Saúde Indígena*).

For second or single doses, no DSEI reached 100% coverage. Pernambuco and Ceará reached 99%, while 16 DSEIs had coverage between 80% and 100%, and 12 between 50% and 70%. The lowest coverage values were observed in Araguaia (44%), Rio Tapajós (40%), and Kaiapó do Pará (34%) (Table 4, Figure 4).

Considering the 90% threshold, 13/34 (38.2%) and 21/34 (61.8%) of DSEIs did not reach the target for first and second or single doses, respectively.

Table 4 also provides contextual information on the number of base centers, villages, ethnic groups, CASAIs, and UBSIs per DSEI. It is important to note that vaccination success is primarily associated with complete vaccination schedules, defined as at least two doses of monovalent vaccines or a single‐dose regimen.

Marked heterogeneity in vaccination coverage was observed across DSEIs and appeared to be closely related to differences in health infrastructure and territorial organization. Districts with larger populations, a higher number of villages, greater ethnic diversity, and wider territorial dispersion, such as Alto Rio Solimões, Yanomami, and Leste de Roraima, consistently exhibited lower vaccination coverage, particularly for complete immunization schedules. These DSEIs are characterized by a high number of remote villages, limited accessibility, and increased dependence on complex logistical operations, including fluvial and aerial transport, which may delay or limit vaccine delivery and administration.

In contrast, DSEIs with more centralized health structures, fewer villages, and greater availability of local health units and support infrastructure, such as Ceará and Pernambuco, achieved the highest vaccination coverage levels, approaching or reaching the 90% threshold recommended by the WHO. These districts typically present more favorable operational conditions, including improved geographic accessibility and more consolidated healthcare networks.

Importantly, the variability observed across DSEIs suggests that vaccination performance is not solely dependent on vaccine availability but is strongly influenced by structural and logistical factors inherent to each territory. Although a formal statistical association was not performed, the descriptive patterns indicate a potential relationship between infrastructure indicators (e.g., number of base centers, villages, and UBSIs) and vaccination outcomes.

Overall, these findings reinforce that geographic isolation, territorial dispersion, and infrastructure limitations play a central role in shaping vaccination coverage among Indigenous populations in Brazil, contributing to persistent regional disparities even in the context of a nationally coordinated immunization program.

### 3.5 Comparative Analysis Between Indigenous and General Population Vaccination Coverage in Brazil

**Figure 5:**
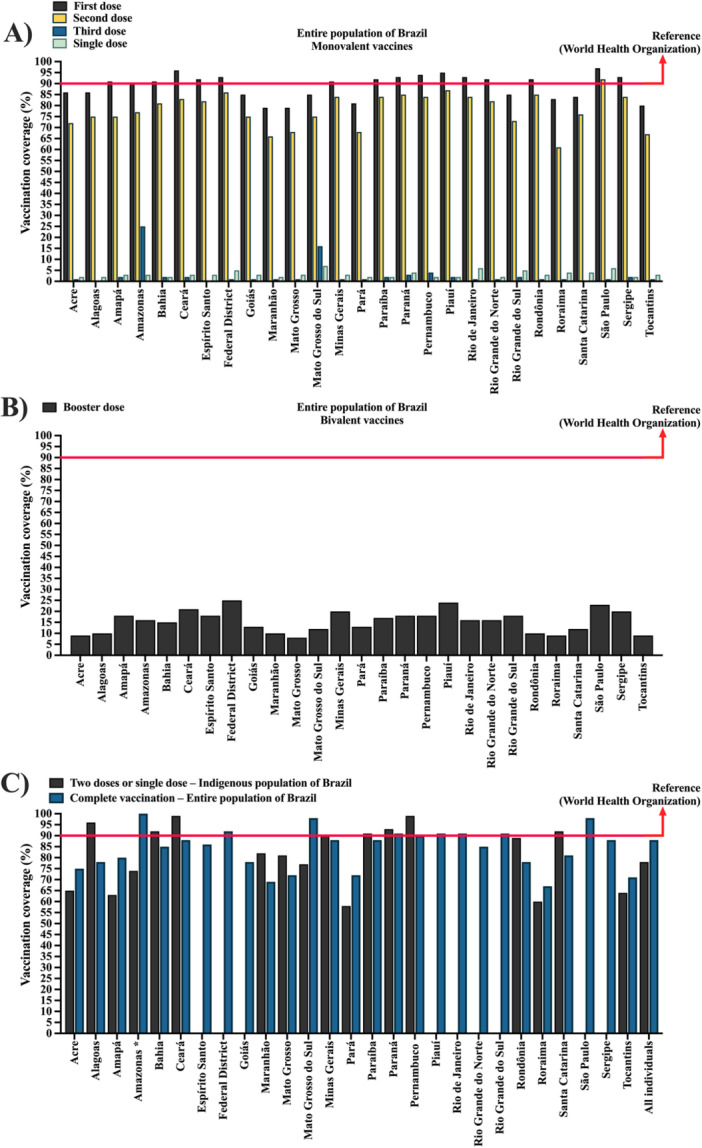
Coronavirus disease 2019 (COVID‐19) vaccination coverage in Brazil across different population groups and vaccine strategies. (A) Panel shows vaccination coverage with monovalent vaccines in the general population, reflecting the initial phase of the immunization campaign. (B) Painel presents coverage with booster doses with bivalent vaccines in the general population, representing updated vaccination strategies targeting emerging variants. (C) Painel focuses on the Indigenous population in Brazil in comparison with entire population, showing coverage by federative unit and considering complete immunization (at least two doses of monovalent vaccines or one single‐dose vaccine). This structure allows comparison between general and Indigenous populations, as well as between different vaccination strategies over time. Some federative units do not have a Special Indigenous Health District (DSEI; acronym for *Distrito Sanitário Especial Indígena*), which may influence regional comparability. Coverage is presented as percentage (%) and calculate as (number of administered doses/Indigenous population) × 100. *, The state of Amazonas showed vaccine coverage above 100%. However, for the figure, coverage values were capped at 100%. Data were obtained from the National Health Data Network (OpenDataSUS), Ministry of Health, Federal Government of Brazil. The data correspond to the period from January 17, 2021, to June 27, 2024. The complete dataset is available at: https://www.gov.br/saude/pt‐br/.

The profile of COVID‐19 vaccination in the general Brazilian population is presented in Table 5 and Figure 5. Among federative units, nine (33%) reached or exceeded 90% coverage for complete vaccination with monovalent vaccines [Amazonas (105%), Mato Grosso do Sul (98%), São Paulo (98%), Federal District (92%), Paraná (91%), Piauí (91%), Rio de Janeiro (91%), Rio Grande do Norte (91%), and Pernambuco (90%)], while several others approached this threshold, indicating a broadly successful primary vaccination rollout at the national level. Overall, first‐dose coverage reached 91%, second‐dose coverage 83%, and complete vaccination 88%, with a national average of 2.6 doses administered per inhabitant.

**Table 5:** Number of doses and vaccination coverage of monovalent coronavirus disease 2019 (COVID‐19) vaccines administered to the Brazilian population.

Federative unit	Population^a^	Doses administered	First dose	Second dose	Third dose	Single dose	Complete vaccination*	Proportion of monovalent vaccines
Acre	830 026	1 758 920	713 114 (86%)	597 499 (72%)	5897 (1%)	19 072 (2%)	622 468 (75%)	2.1
Alagoas	3 127 511	6 879 706	2 688 379 (86%)	2 341 615 (75%)	13 570 (0%)	69 754 (2%)	2 424 939 (78%)	2.2
Amapá	733 508	1 643 530	668 280 (91%)	549 236 (75%)	15 731 (2%)	18 806 (3%)	583 773 (80%)	2.2
Amazonas	3 941 175	9 017 617	3 559 032 (90%)	3 047 051 (77%)	992 976 (25%)	109 173 (3%)	4 149 200 (105%)	2.3
Bahia	14 136 417	36 211 625	12 856 395 (91%)	11 396 394 (81%)	224 733 (2%)	328 375 (2%)	11 949 502 (85%)	2.6
Ceará	8 791 688	23 844 945	8 471 674 (96%)	7 325 866 (83%)	144 333 (2%)	247 451 (3%)	7 717 650 (88%)	2.7
Espírito Santo	3 833 486	9 851 773	3 526 531 (92%)	3 147 864 (82%)	12 257 (0%)	121 018 (3%)	3 281 139 (86%)	2.6
Federal District	2 817 068	7 480 605	2 633 485 (93%)	2 436 606 (86%)	31 232 (1%)	131 063 (5%)	2 598 901 (92%)	2.7
Goiás	7 055 228	15 765 762	6 030 709 (85%)	5 270 632 (75%)	45 325 (1%)	212 954 (3%)	5 528 911 (78%)	2.2
Maranhão	6 775 152	13 363 506	5 359 894 (79%)	4 499 622 (66%)	53 260 (1%)	144 621 (2%)	4 697 503 (69%)	2.0
Mato Grosso	3 658 813	7 147 404	2 894 841 (79%)	2 483 730 (68%)	19 755 (1%)	113 316 (3%)	2 616 801 (72%)	2.0
Mato Grosso do Sul	2 756 700	6 187 719	2 331 322 (85%)	2 064 404 (75%)	441 621 (16%)	200 593 (7%)	2 706 618 (98%)	2.2
Minas Gerais	20 538 718	53 311 664	18 734 237 (91%)	17 165 356 (84%)	239 730 (1%)	693 420 (3%)	18 098 506 (88%)	2.6
Pará	8 116 132	16 073 403	6 614 394 (81%)	5 527 749 (68%)	78 630 (1%)	201 088 (2%)	5 807 467 (72%)	2.0
Paraíba	3 974 495	10 121 607	3 656 421 (92%)	3 327 350 (84%)	85 355 (2%)	74 262 (2%)	3 486 967 (88%)	2.5
Paraná	11 443 208	30 044 688	10 626 061 (93%)	9 702 196 (85%)	318 904 (3%)	432 264 (4%)	10 453 364 (91%)	2.6
Pernambuco	9 058 155	23 383 528	8 542 572 (94%)	7 568 628 (84%)	356 331 (4%)	211 639 (2%)	8 136 598 (90%)	2.6
Piauí	3 269 200	9 227 205	3 096 985 (95%)	2 833 243 (87%)	67 643 (2%)	72 778 (2%)	2 973 664 (91%)	2.8
Rio de Janeiro	16 054 524	42 424 154	14 914 797 (93%)	13 530 564 (84%)	122 007 (1%)	967 478 (6%)	14 620 049 (91%)	2.6
Rio Grande do Norte	3 302 406	8 822 513	3 040 591 (92%)	2 713 787 (82%)	31 995 (1%)	71 611 (2%)	2 817 393 (85%)	2.7
Rio Grande do Sul	10 880 506	28 661 817	10 014 031 (92%)	9 250 847 (85%)	93 666 (1%)	50 090 (5%)	9 847 603 (91%)	2.6
Rondônia	1 581 016	3 310 233	1 349 681 (85%)	1 153 936 (73%)	24 997 (2%)	46 868 (3%)	1 225 801 (78%)	2.1
Roraima	636 303	1 151 909	529 117 (83%)	390 058 (61%)	7684 (1%)	26 866 (4%)	424 608 (67%)	1.8
Santa Catarina	7 609 601	16 853 025	6 407 456 (84%)	5 806 282 (76%)	35 076 (0%)	295 955 (4%)	6 137 313 (81%)	2.2
São Paulo	44 420 459	131 599 420	43 122 197 (97%)	40 852 410 (92%)	278 020 (1%)	2 484 886 (6%)	43 615 316 (98%)	3.0
Sergipe	2 209 558	5 845 804	2 046 292 (93%)	1 848 760 (84%)	40 186 (2%)	46 352 (2%)	1 935 298 (88%)	2.6
Tocantins	1 511 459	2 904 107	1 202 502 (80%)	1 018 770 (67%)	7651 (1%)	43 049 (3%)	1 069 470 (71%)	1.9
Total	203 062 512	522 888 189	185 630 990 (91%)	167 850 455 (83%)	3 788 565 (2%)	7 887 802 (4%)	179 526 822 (88%)	2.6

*Note:* The data correspond to the period from January 17, 2021, to June 27, 2024. The complete dataset is available at: https://www.gov.br/saude/pt‐br/. Data are presented as absolute numbers (*N*) and percentage (%), enabling both quantitative comparison and proportional interpretation. Vaccination coverage was calculated using the formula (number of administered doses/Indigenous population) × 100.

* Complete vaccination (at least two doses of monovalent vaccines or one dose of bivalent vaccine).

^a^ Data were obtained from the National Health Data Network (OpenDataSUS), Ministry of Health, Federal Government of Brazil.

When considering complete vaccination coverage, defined as the receipt of at least two doses of monovalent vaccines or one dose of a single‐dose vaccine, coverage estimates were consistently high across most federative units. In one case, the value exceeded 100% (e.g., Amazonas), reflecting discrepancies in population estimates, population mobility, and vaccination of individuals outside their registered place of residence, rather than true population‐level saturation. Therefore, this indicator should be interpreted cautiously as a proxy for vaccination intensity rather than exact individual‐level coverage.

Supporting Information S1: Table S1 demonstrates that booster doses played a substantial role in the vaccination strategy. First booster doses were widely administered across states (e.g., São Paulo: 67%; Paraná: 57%; Minas Gerais: 54%), whereas second and third booster doses, as well as additional doses, were less frequent, generally remaining below 5%. The uptake of bivalent vaccines was comparatively limited, with a low and relatively homogeneous proportion across federative units (national proportion: 0.2), indicating a gradual transition toward updated vaccination strategies.

In contrast, the vaccination profile observed among Indigenous populations (Table 3) revealed greater heterogeneity. First‐dose coverage ranged from 73% (Pará) to 100% (Ceará), while second‐dose or single‐dose coverage ranged from 58% (Pará) to 99% (Ceará and Pernambuco). Although some states achieved high coverage levels (e.g., Alagoas, Ceará, Pernambuco, and Paraná), others, particularly in the North region, such as Pará and Roraima, showed substantially lower coverage, indicating regional disparities in access and delivery.

Figure 5 integrates these findings across populations and vaccination strategies. Panel A illustrates the high uptake of monovalent vaccines in the general population, reflecting the success of the initial vaccination campaign. Panel B presents the distribution of bivalent booster doses, showing lower overall coverage consistent with the patterns observed in Supporting Table 1. Panel C provides a direct comparison between Indigenous and general populations by federative unit and highlights a persistent disparity. While the general population achieved high and relatively homogeneous levels of complete vaccination, Indigenous populations exhibited more variable and often lower coverage. In several federative units (e.g., Ceará, Pernambuco, and Paraná), Indigenous coverage approached that of the general population; however, in others, particularly Pará and Roraima, the gap was substantial. This pattern indicates that national‐level success in vaccine deployment did not translate uniformly into equitable access for Indigenous communities.

Overall, these findings demonstrate that, despite high national vaccination coverage and substantial vaccine availability, important inequalities persist between population groups. Indigenous populations remain disproportionately affected by lower and more heterogeneous coverage, particularly for complete immunization, reinforcing the need for targeted public health strategies that address geographic, structural, and sociocultural barriers to vaccine delivery.

**Figure 6:**
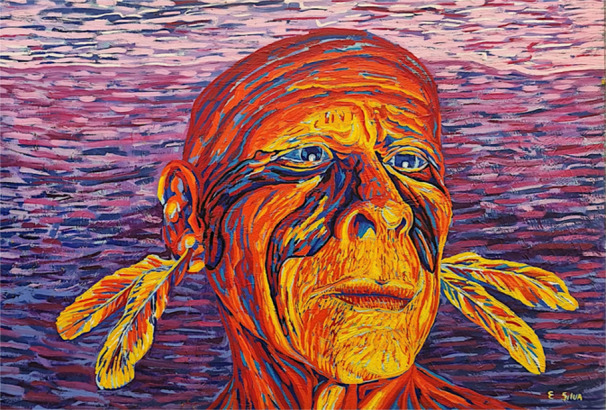
Portrait of an Indigenous person, illustrating cultural representation within the context of the study. Painting by Elvis da Silva (Brazil), oil on canvas.

Figure 6 presents a canvas painting of an Indigenous person by the artist Elvis da Silva.

## 4 Discussion

According to the National Health Data Network, approximately 1.5 million doses of COVID‐19 vaccines were administered to Brazil's Indigenous peoples. Overall, our results indicate that vaccination coverage reached 89% for the first dose, but decreased to 78% for complete vaccination and 43% for booster doses, revealing a substantial drop‐off across the immunization cascade. Among the federative units where the DSEIs are based, there was a great variability in vaccination coverage, with the state of Ceará achieving the best rates, while states such as Pará and Roraima presented some of the lowest complete vaccination coverage levels. Among the 34 DSEIs, 21 did not reach the 90% reference value, related to vaccination success for the first dose, and 13 did not reach the 90% reference value, related to vaccination success for the second dose or single dose. At the district level, vaccination coverage ranged widely, from approximately 56% to 100% for the first dose and from 34% to 99% for complete vaccination, reinforcing marked territorial heterogeneity.

Age‐stratified analyses further demonstrated important disparities, with younger age groups, particularly children aged 3–4 years, showing extremely low vaccination uptake in several DSEIs, whereas older age groups achieved comparatively higher coverage. When compared to the general Brazilian population, Indigenous peoples consistently presented lower complete vaccination rates, despite relatively high initial uptake, indicating persistent inequalities in access to and continuity of care.

These findings are directly supported by our results, which showed an overall first‐dose coverage of 89% and a lower complete vaccination coverage of 78%, highlighting a gap between vaccine initiation and completion across DSEIs.

### 4.1 Underreporting in Indigenous Populations

Although the number of COVID‐19 cases and deaths in Brazil is high, there is evidence of potential underreporting, particularly among the most vulnerable populations [39, 40, 41, 42]. In the context of our findings, this issue is especially relevant, as the marked heterogeneity in vaccination coverage across DSEIs suggests that structural inequalities may also affect epidemiological surveillance and data completeness. Underreporting of COVID‐19 cases may contribute to increased mortality among Indigenous peoples and hinder the appropriate management of cases, including the effective targeting of public health policies for this population [27, 43, 44].

Limited access to healthcare services and delays in seeking of hospital care may further increase mortality, with more severe cases being disproportionately reported. A study assessing clinical symptoms among Indigenous populations during the first year of the pandemic identified oxygen saturation < 95% and respiratory distress as factors associated with an increased risk of death [27], highlighting the need for timely hospital intervention and access to intensive care, particularly in rural areas. DSEIs are responsible for serving multiple ethnic groups across vast and often remote territories, which complicates access to specialized healthcare services. For example, the Alto Rio Juruá DSEI serves 145 villages with only 7 base centers, 1 CASAI, and 1 UBSI. This structural constraint is consistent with our findings, which indicate lower vaccination coverage in DSEIs characterized by greater territorial dispersion and limited healthcare infrastructure. Importantly, these challenges are not unique to Brazil. Indigenous populations in other regions, such as Peru, have also experienced disproportionate impacts from COVID‐19, including higher risks of infection and adverse outcomes [12]. In addition, DSEIs with the lowest vaccination coverage identified in our results, such as Kaiapó do Pará, Rio Tapajós, and Araguaia, may also be more vulnerable to underreporting due to similar structural barriers.

### 4.2 Scientific Production on Indigenous Health

Despite the growing awareness of the vulnerability of Indigenous peoples during the COVID‐19 pandemic, scientific production on this topic remains limited. A recent study revealed that, of 153 publications on Indigenous peoples and COVID‐19, only 18% focused specifically on incidence, 16% on mortality, 12% on mental health, and 0.04% on the diagnosis of COVID‐19, thus highlighting the scarcity of research dedicated to their reality [45]. This scenario reflects social invisibility, historical marginalization, socio‐cultural loss, and loss of vital data, not only for the Indigenous population, but for the entire national identity [2]. This invisibility is reflected in our findings, which demonstrated substantial disparities in vaccination coverage across DSEIs, ranging from 56% to 100% for the first dose and from 34% to 99% for complete vaccination. Although Brazil is one of the countries with the highest number of publications on the subject, with 18% of peer‐reviewed articles focusing on its Indigenous population, the overall volume of research remains disproportionately low, reflecting a significant gap in the academic literature on health and the impacts of the pandemic on Indigenous communities [45]. Furthermore, in Brazil, the incidence and mortality of COVID‐19 were, respectively, 136% and 110% higher among Indigenous peoples compared to the national average [39]. These epidemiological disparities are consistent with our observation that, despite relatively high first‐dose coverage (89%), complete vaccination coverage remained lower (78%), suggesting persistent inequities in access and continuity of care.

### 4.3 Determinants of Vaccination Coverage Adherence in Indigenous Communities

Indigenous peoples have worse health conditions compared to the rest of the population of Brazil [28, 46]. This is largely due to the colonization process, which has disrupted traditional ways of life, led to the loss of territories, environmental degradation, racial discrimination, and socio‐economic and political marginalization. In a study that assessed reported cases of Indigenous people with severe acute respiratory syndrome in Brazil during the first year of the COVID‐19 pandemic, a total of 3122 cases were reported, of which, 1994 were diagnosed with COVID‐19 and 40.2% progressed to death [27]. In addition, another study evaluated 3062 cases of Indigenous people with severe acute respiratory syndrome in Brazil in 2020. Cases and deaths were concentrated in the states of Amazonas and Mato Grosso do Sul, located in the North and Central‐West regions of Brazil. In this study, the highest risk of death occurred among the elderly [OR = 6.29 (95% CI = 4.71–8.39)], those with low levels of education [OR = 1.72 (95% CI = 1.22–2.28)], those living in rural areas [OR = 1.35 (95% CI = 1.12–1.62)], and those with comorbidities [OR = 1.87 (95% CI = 1.42–2.46)], especially obesity [OR = 2.56 (95% CI = 1.07–6.11)] [31]. Another study investigating the public health emergency among the Yanomami Indigenous population between 2018 and 2022 reported that this group had an infant mortality rate approximately 1.5–3.5 times higher than that of the country's total Indigenous population [26]. These data reinforce the need to expand vaccination among Indigenous populations, as observed in this study, which showed low vaccination coverage rates in numerous DSEIs. In line with our findings, 13 out of 34 DSEIs (38.2%) did not reach the 90% target for first‐dose coverage, and 21 (61.8%) did not reach this threshold for complete vaccination, reinforcing the impact of these vulnerabilities. Vaccination, in this context, is crucial for controlling the pandemic and for preventing deaths among older Indigenous individuals – key custodians of cultural knowledge and traditions within their communities [2].

Deforestation, land grabbing, and mining on Indigenous lands have significantly exacerbated the vulnerability of Indigenous peoples to COVID‐19, contributing to an increase in cases of diseases, including COVID‐19 [47, 48, 49, 50, 51, 52]. This scenario is intensified by the invasion of illegal miners and loggers, who not only increase exposure to the virus but also deteriorate the socio‐environmental conditions of these regions [48], as the spread of the coronavirus has been facilitated by these activities, which have internalized, via roads and waterways, the spread of the disease in areas that were previously less connected, such as the Yanomami territories [48]. In addition, deforestation and mining have created environmental stressors that have worsened the health of Indigenous peoples and facilitated the spread of the coronavirus [39]. These contextual factors are consistent with our observation that DSEIs located in regions with greater environmental and territorial vulnerability tended to show lower vaccination coverage.

In Brazil, the lack of communication strategies on COVID‐19 prevention has represented a significant challenge during the pandemic, as the absence of accessible information materials in Indigenous languages complicated the dissemination of prevention and vaccination guidelines, leaving these communities more vulnerable to the virus [45]. In response to this gap, Indigenous peoples have used social media platforms, such as Instagram, to raise awareness of rights violations and mobilize organized forms of resistance. These platforms have thus acted as a means for collective fundraising, supporting legal initiatives against the government due to the lack of adequate support during the health crisis [45]. However, it is important to note that social media has also been used to disseminate negative discourse against these populations, intensifying the challenges they face and highlighting the need for continuous monitoring and protective policies. It should also be noted that evaluating the effectiveness of these communication strategies is crucial to ensuring that messages are delivered in a clear and culturally appropriate manner, promoting the health and safety of Indigenous populations in Brazil [45]. These communication barriers are reflected in our findings, particularly in DSEIs with lower vaccination uptake and slower progression to complete vaccination schedules.

### 4.4 Contributions of Vaccines to Indigenous Health

Vaccination is one of the most effective public health strategies, and Brazil has historically demonstrated strong immunization capacity. In 2010, during the H1N1 pandemic, Brazil became a global reference in immunization, vaccinating 88 million people in 3 months, corresponding to nearly 45% of the population. In the recent SARS‐CoV‐2 pandemic, the scenario was quite different: in 3 months of campaigning, the country vaccinated just over 25 million people with the first dose (12% of the population) and 8.5 million (4% of the population) with the second dose [53]. This discrepancy has been largely attributed to delays in vaccine procurement by the federal government [54].

Within this national context, the organization of Indigenous healthcare plays a central role in shaping vaccination outcomes. The DSEIs coordinate care for the Indigenous population and are responsible for vaccination and healthcare programs [55]. They operate under the responsibility of the SESAI and are territorially defined based on epidemiological, geographical, and ethnographic criteria [56]. The DSEIs' approach is based on a decentralized model of care that involves co‐responsibility between Indigenous communities and SESAI, including UBSIs located on Indigenous lands that provide primary health care [56].

To guarantee medical care in more isolated communities, there are itinerant teams made up of Indigenous health agents and non‐Indigenous health professionals, which are essential for reaching geographically remote populations. For cases that require more complex care, the DSEIs have CASAIs, which are housing units located in urban areas [57]. In the CASAI, Indigenous patients and their families can stay during treatment at secondary or tertiary care levels. This arrangement facilitates access to specialized medical procedures that cannot be performed within Indigenous territories, expanding the scope of care available to these populations.

A study conducted in 2011 in the DSEI of Alto do Rio Negro sought to analyze the health practices developed there, with special attention to the Baniwa ethnic group in the Northwest of the Brazilian Amazon, as well as the conceptions and social practices structured around vaccination [58]. The study described how, due to the great distances between the municipal headquarters and villages, together with the lack of electricity and supplies to preserve immunobiologicals, vaccination activities are primarily carried out on a campaign basis. This logistical limitation reinforces dependence on periodic vaccination strategies rather than continuous immunization delivery. The study also mentions that support sites with health posts equipped with freezers powered by generators can be found within Indigenous areas, allowing vaccines to be stored and ice to be replenished, even if only for a short time [58].

The healthcare team's means of communication with Indigenous communities was based on the use of radios installed in some locations, which were occasionally interrupted by power outages, preventing the transmission of information to community members. Such communication gaps contribute to poor synchronization between healthcare teams and community members, limiting vaccination adherence. Those who were absent on the day of vaccination could only receive the dose on subsequent visits, commonly carried out months later. Conversely, healthcare teams could arrive late or fail to appear on the scheduled date due to difficult travel routes in the Amazon, reducing the number of Indigenous people available for vaccination. These operational challenges may also generate tensions between healthcare providers and Indigenous communities, particularly when activities cannot be easily rescheduled [58].

In addition, another important factor to consider regarding vaccination in Indigenous populations is their understanding of the disease process. The author explains that the Indigenous perspective on illness is, above all, plural: a collective threat that requires equally collective therapeutic strategies, such as shamanism. This perspective may diverge from the individual‐centered biomedical model, potentially influencing engagement with vaccination programs [58]. Even though the article does not deal with the COVID‐19 pandemic period, it serves as a basis for delving deeper into the issue of the country's Indigenous population. These findings remain relevant, as structural barriers such as infrastructure limitations, logistical constraints, and communication failures continue to affect vaccination coverage across different DSEIs. Conditions marked by power outages and lack of synchronization between healthcare teams and Indigenous populations may lead to reduced vaccination adherence throughout the Brazilian territory. In addition, each DSEI has a defined geographical location and population size, and this heterogeneity should be considered when allocating resources and planning vaccination strategies. Although the article deals with the Alto do Rio Negro DSEI, which obtained complete vaccination coverage during the COVID‐19 pandemic, it is possible to apply this reasoning to other DSEIs that have obtained unsatisfactory vaccination coverage rates [58]. Importantly, geographic remoteness alone does not fully explain disparities, as some hard‐to‐reach DSEIs have achieved high vaccination coverage [58]. In addition, the 9‐year gap between the study and the pandemic may have introduced changes, such as greater use of communication technologies and increased awareness of health‐related process among both Indigenous populations and healthcare professionals.

Vaccines are safe and extremely important for reducing severe symptoms and worse outcomes [44, 59], but due to socio‐economic and biological issues, vulnerable groups such as the Indigenous population can be seriously affected by delays or lack of vaccination [9]. The COVID‐19 vaccination campaign reached approximately 90% coverage for the first dose and 85% for the second dose by December 2021; however, significant disparities persisted across DSEIs, particularly in the pace of vaccine rollout [24].

Vaccines are generally acceptable tools among the Indigenous population. An example of this is the influenza (H1N1) campaign in 2020, which achieved 94% coverage among Indigenous individuals over 6 months of age within 3‐months. However, the COVID‐19 vaccination campaign produced different results [60]. One factor that strongly influenced this was the spread of digital misinformation, which reached the Indigenous territories and generated fear and uncertainty [61, 62, 63, 64]. The lack of culturally appropriate educational materials in Indigenous languages further contributed to low initial vaccination uptake [62], while reports of deaths temporally associated with vaccination increased insecurity, despite no causal relationship. Faced with this scenario, targeted communication strategies have been implemented, including the development of culturally adapted materials such as booklets and podcasts. These initiatives aim to demonstrate that traditional Indigenous medicine and biomedical approaches, including vaccination, are not mutually exclusive but can be complementary, thereby strengthening trust and improving vaccination adherence [60].

During the COVID‐19 pandemic, an important spokesperson for Indigenous peoples in Brazil was Sonia Guajajara who served as the executive coordinator of the Articulation of Indigenous Peoples of Brazil (APIB; acronym for *Articulação dos Povos Indígenas do Brasil*). The organization played a leading role in COVID‐19 monitoring, communication, and surveillance strategies. Among its initiatives was the “Parente Vaccine” campaign, which, in response to the rising number of deaths, proactively advocated for the prioritization of Indigenous peoples in the national vaccination strategy. Furthermore, once vaccination began, Guajajara acted as a key spokesperson in combating misinformation [65].

Overcoming communication challenges and respecting cultural beliefs in Indigenous contexts require translating theoretical frameworks into culturally adapted, practical health communication strategies, as demonstrated by successful experiences in Brazil [66, 67]. The e‐COVID Xingu project exemplifies this approach by integrating social media and podcasts with the production of bilingual educational materials (Portuguese and Kayapó), thereby ensuring that that technical information is both accessible and respectful of linguistic diversity [66].

Beyond content, the choice of communication medium represents a critical practical consideration. For instance, the use of Radio 93.1 FM in the Trans‐Amazonian region proved to be an effective strategy for reaching populations with limited internet connectivity, highlighting that cultural adaptation must also encompass technological appropriateness to the local context [67].

An adapted approach and culturally appropriate communication about vaccination directed toward Indigenous peoples are necessary, as there is often a cultural understanding that may generate apprehension or limited knowledge regarding its effects. In the passage by Marcos Pellegrini (1993, apud Garnelo, 2011), based on his contact with the Yanomami, he reports the following interaction [58]:

“The people of the mountains did not know the liquids that live in the cold‐water tanks and that drive away epidemics, just like many nabèbè, who also do not know or doubt their effects and who make the body bitter so that the xawala does not take it. And there were liquids for various types of xawalabè, except for the one that was arriving the most—the one they call flu. And people were afraid of injections, but much more afraid of the xawalabè, and so they accepted the pricks… many and repeated… although the xawala flu always arrived”

(p. 52).

### 4.5 Logistics and Distribution of Vaccinations in Indigenous Populations

The Indigenous population experiences persistent vaccination disparities worldwide, with consistently lower coverage reported across different settings. In Mexico, Indigenous language speakers have shown lower vaccination rates compared to non‐Indigenous populations, with barriers such as language and lack of civil documentation limiting access to health services [68, 69]. Similar patterns of hesitancy have been linked to misinformation, as observed among Native Hawaiians during the COVID‐19 pandemic [70]. In the Philippines, vaccination intention among the Dumagat Remontado people have been associated with public policies, perceived disease severity, and risk–benefit understanding [71]. Conversely, evidence from Quebec, Canada, highlights that the presence of Indigenous healthcare professionals can increase trust and improve vaccine uptake [72]. Together, these findings reinforce that structural barriers, communication challenges, and trust‐building measures are central determinants of vaccination coverage, factors that are also highly relevant to the Brazilian Indigenous context.

Regarding vaccine distribution, the Ministry of Health only approved vaccination for Indigenous adolescents 4 months after the National Health Surveillance Agency had approved it for the 12‐ to 17‐year‐old population [60]. It is important to note that, as this age group represents a large portion of the population, the number of cases increased. At the end of October 2021, for example, COVID‐19 cases rose sharply in schools in the Dourados Indigenous Reserve (Mato Grosso do Sul), leading to the temporary suspension of classes. In addition, between October 17 and November 20, 2021, 226 new cases of COVID‐19 were registered in the DSEI of Mato Grosso do Sul, which, until then, represented the highest number among the 34 DSEIs [60]. There was also a logistical challenges, as the Pfizer vaccine (BNT162b2), which received full authorization for use in the country on February 3, 2021 [73], had a shelf life of only 31 days in standard refrigerators. Thus, the pandemic highlighted factors such as the social vulnerability of Indigenous populations and the precariousness of health, housing, and education services [62].

The Ministry of Health's SESAI clarified on the Repórter Brasil website that when the first vaccine doses against COVID‐19 arrived in Brazil on January 18, 2021, Indigenous health services received one‐third of the vaccines, corresponding to the amount required for the first and second doses for Indigenous individuals aged ≥ 18 and Indigenous health workers across the 34 DSEIs [9, 74, 75]. This indicates a clear disproportion between the acquisition and production of vaccines by the Brazilian government and the national demand for vaccination, especially among priority groups such as Indigenous peoples. SESAI also reported that, as of December 8, 2021, 89% of the Indigenous population aged ≥ 18 had received the first dose of the COVID‐19 vaccine and 83% had received the second dose, according to the National Plan for the Operationalization of Vaccination against COVID‐19 [74]. However, among those with more severe clinical vulnerability, as of March 1, 2022, only 48.7% of the Indigenous population over 20 years of age had been fully vaccinated under the care of the DSEIs. This rate was 30% lower than that of the non‐Indigenous population, which had 74.8% vaccination coverage [25]. These findings suggest lower vaccination coverage among the adult Indigenous population compared to the general population in Brazil [24, 25]. Vaccination coverage among children aged 5 and 9 years was even more concerning. By the same date, only 2.6% of the Indigenous population in this age group within the DSEIs had received their first dose, whereas the national average was 40.7% [25].

An analysis by the Institute of Socioeconomic Studies showed that spending on Indigenous health decreased by 9% in the first 6 months of 2020, during the pandemic. The budget fell from 725.9 million reais in the first half of 2019 to 708.8 million in the same period of 2020. In April and May, when COVID‐19 began to threaten even isolated populations, the amounts allocated to Indigenous health were lower than those invested in 2019: they decreased from 236.4 million to 173.3 million reais in April and from 159.2 million to 54.8 million reais in May [76, 77].

The low vaccination coverage observed among Indigenous population in Brazil is likely influenced by a combination of factors, including: (a) limited access to health services, as many Indigenous communities are located in remote areas, making it difficult to access vaccines and immunization‐related information; (b) vaccination logistics, since the distribution and administration of vaccines in isolated areas are complex and often inefficient; and (c) cultural and linguistic diversity, as differences among ethnic groups can hinder effective communication about the importance of vaccination.

These findings are consistent with evidence from the literature indicating that additional factors also play a role, including: (a) distrust of institutions, particularly toward government and health systems, stemming from a history of neglect and rights violations, which may contribute to resistance to vaccination; and (b) lack of information, as the spread of misinformation and the absence of culturally adapted awareness campaigns have contributed to vaccine hesitancy [2, 9, 24, 75, 78, 79, 80, 81]. Together, these factors pose significant challenges to achieving high vaccination coverage in Indigenous communities.

### 4.6 Implementation Strategies for Indigenous Vaccination Programs

To provide better vaccination coverage among Indigenous people in Brazil, a number of strategies can be implemented, including: (a) access to health services: strengthening the health infrastructure in communities, ensuring that vaccines reach remote areas; (b) awareness campaigns: developing information campaigns that respect local cultures and languages, using community leaders and Indigenous representatives to promote vaccination; (c) health education: providing health education that addresses myths and misinformation about vaccines, emphasizing their importance for individual and collective protection; (d) community participation: involving Indigenous communities in the planning and execution of vaccination programs, respecting their traditions and ways of life; (e) efficient logistics: improving vaccine distribution logistics, ensuring that doses are delivered properly and on time; (f) partnerships with Indigenous organizations: collaborating with Indigenous organizations and associations to facilitate communication and the implementation of vaccination strategies; and (g) monitoring and evaluation: conducting continuous follow‐up of vaccination coverage and community reactions, adjusting approaches as necessary [9, 45, 75, 78, 80, 82, 83, 84, 85]. These actions can help increase confidence in vaccines and improve vaccination coverage among Indigenous peoples.

In summary, improving vaccination uptake among Indigenous populations in Brazil requires a coordinated and sustained strategy centered on four key priorities. First, strengthening primary healthcare infrastructure within the DSEIs is essential to ensure consistent access to immunization services, adequate logistics, and continuity of care in geographically dispersed territories. Second, expanding the training and sustained support of Indigenous health professionals is critical, as these workers play a central role in mediating care, fostering trust, and ensuring that health interventions are culturally appropriate and locally accepted. Third, co‐developing culturally grounded communication strategies in partnership with Indigenous leadership can enhance the effectiveness of public health messaging by aligning it with local languages, belief systems, and community dynamics, thereby reducing misinformation and increasing vaccine confidence. Finally, scaling up mobile vaccination teams is fundamental to overcoming geographic and structural barriers, enabling timely and equitable access to vaccines in remote and hard‐to‐reach areas. Together, these integrated actions highlight the importance of combining structural investments, workforce development, culturally responsive communication, and adaptive service delivery models to promote more equitable and effective vaccination strategies in Indigenous contexts.

### 4.7 Comparison With Other Vulnerable Populations, Including Indigenous Groups

To contextualize the findings of this study at the national level, Costa et al. (2025) investigated COVID‐19 vaccination coverage in Quilombola communities, showing rates below 50% for both the first dose (48.4%) and the second or single dose (49.4%), with an average of 159.26 doses administered per 100 inhabitants [86]. There was wide variability across regions, and vaccination was positively correlated with the Human Development Index (HDI) as well as with the quality of health services. However, higher municipal health expenditures showed a negative correlation, suggesting that the mere allocation of resources does not guarantee vaccination success.

In parallel, a cohort study involving 389 753 Indigenous individuals showed a complete vaccination rate of only 48.7%, compared to 74.8% in the general population, indicating significant inequality in vaccination coverage and establishing a pattern of low coverage among vulnerable populations [87].

Therefore, both in Quilombola and Indigenous contexts, low vaccination coverage is not solely due to vaccine availability but also results from the interaction between socioeconomic vulnerability (reflected by low HDI and fragile infrastructure) and operational limitations in health services. In the case of the DSEIs, the structural precariousness of UBSIs and inefficient information management are factors that exacerbate this inequality. These parallels highlight that reducing disparities requires actions that strengthen both the socioeconomic dimension and the operational capacity of primary health care [40].

In general, Indigenous peoples in Brazil have been disproportionately affected by COVID‐19 for several interconnected reasons, including: (a) limited access to health services: many Indigenous communities live in remote areas where access to care is precarious, hindering prevention and treatment; (b) living conditions: in many communities, overcrowded housing and a lack of basic sanitation facilitate the spread of the virus; (c) socioeconomic vulnerability: Indigenous peoples often face inequalities that increase susceptibility to complications from COVID‐19, including pre‐existing conditions; (d) racial and historical inequality: the history of marginalization and discrimination contributes to increased vulnerability during public health crises; and (e) impacts of territorial exploitation: the invasion of Indigenous lands for activities such as mining and farming increases contact with pathogens and introduce new diseases into communities [4, 45, 75, 78, 88, 89, 90, 91]. These factors, among others, contributed to higher infection and mortality rates among Indigenous peoples during the pandemic. Our study reinforces this pattern by showing that Indigenous populations experienced lower and more heterogeneous vaccination coverage compared to the general population.

## 5 Limitations

During the study, several important limitations were identified and should be more explicitly considered when interpreting the findings. First, the analyses relied on administrative vaccination records from official databases, without the possibility of independent validation. This may introduce uncertainties related to data accuracy, completeness, and potential misclassification, particularly regarding Indigenous identification.

In addition, the platform does not provide sex‐disaggregated data (male and female) for Indigenous individuals vaccinated against COVID‐19, making only the total number of vaccinated individuals available. Regarding the distribution of administered doses across federative units, only the percentage of first and second or single doses are reported, without specification of which DSEIs compose each unit. This restricts a more detailed and critical assessment of vaccination coverage at subnational levels.

For age‐stratified data, the platform reports the number of doses administered without linking these figures to the corresponding population size in each age group, thereby preventing the calculation of age‐specific vaccination coverage. Similarly, there is no detailed characterization of vaccine types, which precludes the identification of individuals who received single‐dose vaccines within categories that combine second and single doses.

Furthermore, the analyses are based on aggregated secondary data, without access to individual‐level records. As a result, it was not possible to assess data completeness, identify missing information, or apply statistical methods to address incomplete data. Importantly, the study did not allow adjustment for relevant confounding factors, such as geographic remoteness, access to healthcare services, and socioeconomic conditions, which may significantly influence vaccination coverage.

It is also important to highlight that the denominator used to estimate vaccination coverage was based on population data from IBGE, which may underestimate Indigenous populations and includes individuals who self‐identify as Indigenous regardless of residence in Indigenous territories. In contrast, vaccination data are derived from the SASI‐SUS, organized through DSEIs, whose registered population reflects an ascribed group defined by healthcare linkage and ethno‐cultural criteria. Potential discrepancies between these population bases should therefore be considered when interpreting coverage estimates.

Moreover, despite the availability of a multi‐year dataset, the study did not include a detailed temporal analysis of vaccination trends over time, limiting the ability to assess changes in coverage dynamics throughout the pandemic.

Finally, due to the structure of the database and the organization of DSEIs, it was not possible to evaluate population mobility, such as movement between Indigenous lands and urban areas during the pandemic. These dynamics are not captured in the available data and could not be incorporated into the analyses.

Despite these limitations, the study is based on an official nationwide database maintained by the Brazilian Ministry of Health, which represents the primary source of epidemiological information in the country. In addition, the large population base covered contributes to the robustness of the findings, although the aforementioned limitations should be carefully considered when interpreting the results.

## 6 Conclusion

Vaccination coverage against COVID‐19 among Indigenous populations in Brazil has not yet reached the 90% target recommended by the Brazilian government in alignment with the WHO, after 4 years of the onset of the pandemic. Among the 34 DSEIs, only 21 achieved this threshold for the first dose, and just 13 reached it for the second or single dose. These findings reinforce the persistent gaps in vaccination completion, and well as marked regional inequalities in coverage. Taken together, these results underscore the need to strengthen and better coordinate public health policies aimed at Indigenous populations. Efforts should prioritize expanding and sustaining primary healthcare infrastructure within DSEIs, particularly in regions with lower vaccination coverage, to ensure equitable access to immunization services. In parallel, greater investment in the training and long‐term support of Indigenous health professionals is essential, given their key role in fostering trust and facilitating culturally appropriate care. Furthermore, improving vaccination uptake requires the development of culturally responsive strategies that are co‐created with Indigenous leadership and communities. Approaches that respectfully engage with traditional knowledge systems, while aligning with evidence‐based public health practices, may contribute to reducing misinformation and increasing vaccine confidence. Finally, adaptive service delivery models, including the expansion of mobile vaccination teams, are critical to overcoming geographic barriers and reaching remote and underserved populations. Overall, advancing equitable vaccination coverage in Indigenous contexts depends on integrated actions that combine structural investment, community engagement, and culturally grounded health strategies, thereby enhancing both the effectiveness and sustainability of immunization programs.

## Author Contributions

[N.M.S.S., L.S.M., and F.A.L.M.] collected and tabulated the data. [N.M.S.S., P.T.C., L.S.M., L.F.A.M., V.S.dos.S., and F.A.L.M.] interpreted the study findings. [N.M.S.S., P.T.C., L.S.M., L.F.A.M., V.S.dos.S., and F.A.L.M.] drafted and critically revised the manuscript. [N.M.S.S., P.T.C., L.S.M., L.F.A.M., V.S.dos.S., and F.A.L.M.] approved the final version of the manuscript and agreed to its submission.

## Ethics Statement

The authors have nothing to report.

## Conflicts of Interest

The authors declare no conflicts of interest.

## Supporting information

Supporting File

## Data Availability

The material used in this study are available upon request from the corresponding author and are also publicly accessible through the Brazilian Ministry of Health's platform, the coronavirus disease 2019 (COVID‐19) vaccines database (https://infoms.saude.gov.br/).
