# Supplementary material for: Vaccination Coverage Against Coronavirus Disease 2019 (COVID‐19) in Brazil's Indigenous Population
Source: J Med Virol. 2026 May 18;98:e70965. doi: 10.1002/jmv.70965 (PMC13182200; doi:10.1002/jmv.70965)
Supplement: Supplementary file 1 — Supporting File [file JMV-98-e70965-s001.docx]

**Supplementary Material**

**Title:** Vaccination Coverage Against Coronavirus Disease 2019 (COVID-19) in Brazil’s Indigenous Population

**Short title:** Indigenous Population and Vaccination

Nathália Mariana Santos Sansone ^1,2,3,#^; Patrícia Teixeira Costa ^1,2,3,#^; Lucas Silva Mello ^1,2,3,#^; Luiz Felipe Azevedo Marques ^1,2,3,#^; Vinícius Santiago dos Santos ^1,2,3,#^; Fernando Augusto Lima Marson ^1,2,3,*,#^

^1^ Laboratory of Molecular Biology and Genetics, Postgraduate Program in Health Sciences, Postgraduate Program in Health Data Science, São Francisco University (USF; acronym for *Universidade São Francisco*), Bragança Paulista, São Paulo, Brazil.

^2^ Laboratory of Clinical and Molecular Microbiology, Postgraduate Program in Health Sciences, Postgraduate Program in Health Data Science, São Francisco University (USF; acronym for *Universidade São Francisco*), Bragança Paulista, São Paulo, Brazil.

^3^ LunGuardian Research Group – Epidemiology of Respiratory and Infectious Diseases, Postgraduate Program in Health Sciences, Postgraduate Program in Health Data Science, São Francisco University (USF; acronym for *Universidade São Francisco*), Bragança Paulista, São Paulo, Brazil.

*** Corresponding Author:** [FALM] Fernando Augusto Lima Marson, BSc, MSc, PhD.

Laboratory of Molecular Biology and Genetics, Laboratory of Clinical and Molecular Microbiology, LunGuardian Research Group – Epidemiology of Respiratory and Infectious Diseases, Postgraduate Program in Health Sciences, Postgraduate Program in Health Data Science, São Francisco University (USF; acronym for *Universidade São Francisco*). Avenida São Francisco de Assis, 218. Jardim São José, Bragança Paulista 12916-900, São Paulo, Brasil. Phone: +55-19-999752911.

**E-mail:**  fernando.marson@usf.edu.br and fernandolimamarson@hotmail.com

^#^ The authors contributed equivalently to the study.

| **Supplementary Table 1.** Distribution and proportion of monovalent and bivalent coronavirus disease 2019 (COVID-19) vaccine doses administered across Brazilian Federative Units. ^a^ | | | | | | | | | |
| --- | --- | --- | --- | --- | --- | --- | --- | --- | --- |
| **Federative unit** | **Monovalent vaccines** | | | | | **Bivalent vaccines** | | | |
|  | **Booster dose** | **1^st^ booster dose** | **2^st^ booster dose** | **3^st^ booster dose** | **Additional dose** | **Total bivalent doses administered** | **Booster dose** | **Booster other doses** | **Proportion of bivalent vaccines** |
| Acre | 297 942 (36%) | 6083 (1%) | 94 092 (11%) | 1271 (0%) | 22 653 (3%) | 81 294 | 77 241 (9%) | 4053 (0%) | 0.1 |
| Alagoas | 1 277 030 (41%) | 26 073 (1%) | 446 848 (14%) | 2883 (0%) | 11 642 (0%) | 324 537 | 323 585 (10%) | 952 (0%) | 0.1 |
| Amapá | 263 735 (36%) | 19 904 (3%) | 79 300 (11%) | 869 (0%) | 26 309 (4%) | 130 848 | 128 653 (18%) | 2195 (0%) | 0.2 |
| Amazonas | 834 689 (21%) | 141 562 (4%) | 302 763 (8%) | 2572 (0%) | 22 140 (1%) | 653 560 | 648 138 (16%) | 5422 (0%) | 0.2 |
| Bahia | 7 356 387 (52%) | 87 862 (1%) | 3 432 390 (24%) | 58 249 (0%) | 460 214 (3%) | 2 130 516 | 2 102 861 (15%) | 27 655 (0%) | 0.2 |
| Ceará | 4 698 990 (53%) | 314 502 (4%) | 2 277 132 (26%) | 116 569 (1%) | 242 175 (3%) | 1 830 210 | 1 809 709 (21%) | 20 501 (0%) | 0.2 |
| Espírito Santo | 1 887 580 (49%) | 67 842 (2%) | 974 721 (25%) | 25 470 (1%) | 88 121 (2%) | 702 520 | 693 040 (18%) | 9480 (0%) | 0.2 |
| Federal District | 1 479 499 (53%) | 50 779 (2%) | 644 018 (23%) | 6655 (0%) | 63 689 (2%) | 699 905 | 697 342 (25%) | 2563 (0%) | 0.2 |
| Goiás | 2 830 802 (40%) | 40 003 (1%) | 1 107 354 (16%) | 17 328 (0%) | 206 636 (3%) | 918 059 | 903 269 (13%) | 14 790 (0%) | 0.1 |
| Maranhão | 2 218 100 (33%) | 35 862 (1%) | 847 994 (13%) | 54 505 (1%) | 146 357 (2%) | 721 124 | 711 363 (10%) | 9761 (0%) | 0.1 |
| Mato Grosso | 1 147 643 (31%) | 10 139 (0%) | 360 780 (10%) | 10 836 (0%) | 105 636 (3%) | 283 689 | 277 606 (8%) | 6083 (0%) | 0.1 |
| Mato Grosso do Sul | 854 985 (31%) | 8584 (0%) | 216 251 (8%) | 5952 (0%) | 60 963 (2%) | 342 135 | 337 377 (12%) | 4758 (0%) | 0.1 |
| Minas Gerais | 11 080 795 (54%) | 112 788 (1%) | 4 298 321 (21%) | 60 996 (0%) | 914 774 (4%) | 4 085 125 | 4 063 788 (20%) | 21 337 (0%) | 0.2 |
| Pará | 2 483 551 (31%) | 75 121 (1%) | 800 324 (10%) | 57 267 (1%) | 228 187 (3%) | 1 030 845 | 1 017 335 (13%) | 13 510 (0%) | 0.1 |
| Paraíba | 2 130 549 (54%) | 52 684 (1%) | 731 022 (18%) | 4395 (0%) | 58 547 (1%) | 662 075 | 657 208 (17%) | 4867 (0%) | 0.2 |
| Paraná | 6 516 935 (57%) | 144 956 (1%) | 1 816 106 (16%) | 18 472 (0%) | 459 532 (4%) | 2 024 832 | 2 017 604 (18%) | 7 228 (0%) | 0.2 |
| Pernambuco | 4 540 661 (50%) | 80 470 (1%) | 1 653 127 (18%) | 285 731 (3%) | 140 229 (2%) | 1 721 274 | 1 645 531 (18%) | 75 743 (1%) | 0.2 |
| Piauí | 1 980 215 (61%) | 59 869 (2%) | 1 068 186 (33%) | 5662 (0%) | 39 531 (1%) | 795 562 | 787 674 (24%) | 7888 (0%) | 0.2 |
| Rio de Janeiro | 8 399 781 (52%) | 60 672 (0%) | 3 943 613 (25%) | 71 078 (0%) | 404 166 (3%) | 3 111 614 | 3 087 022 (19%) | 24 592 (0%) | 0.2 |
| Rio Grande do Norte | 1 747 501 (53%) | 42 934 (1%) | 854 455 (26%) | 154 286 (5%) | 164 262 (5%) | 528 338 | 514 134 (16%) | 14 204 (0%) | 0.2 |
| Rio Grande do Sul | 5 893 869 (54%) | 59 495 (1%) | 2 553 234 (23%) | 17 948 (0%) | 269 949 (2%) | 1 988 763 | 1 978 966 (18%) | 9797 (0%) | 0.2 |
| Rondônia | 510 103 (32%) | 3824 (0%) | 169 060 (11%) | 2679 (0%) | 48 282 (3%) | 167 848 | 162 157 (10%) | 5691 (0%) | 0.1 |
| Roraima | 143 451 (23%) | 5570 (1%) | 37 239 (6%) | 849 (0%) | 10 168 (2%) | 63 855 | 59 775 (9%) | 4080 (1%) | 0.1 |
| Santa Catarina | 3 085 443 (41%) | 49 821 (1%) | 1 053 408 (14%) | 13 261 (0%) | 100 304 (1%) | 897 208 | 877 039 (12%) | 20 169 (0%) | 0.1 |
| São Paulo | 29 698 771 (67%) | 964 085 (2%) | 13 619 862 (31%) | 23 137 (0%) | 533 631 (1%) | 10 402 691 | 10 365 377 (23%) | 37 314 (0%) | 0.2 |
| Sergipe | 1 193 788 (54%) | 15 782 (1%) | 603 638 (27%) | 22 362 (1%) | 26 695 (1%) | 443 584 | 441 162 (20%) | 2422 (0%) | 0.2 |
| Tocantins | 462 845 (31%) | 7056 (0%) | 131 610 (9%) | 2660 (0%) | 26 017 (2%) | 140 840 | 138 511 (9%) | 2329 (0%) | 0.1 |
| Total | 105 015 640 (52%) | 2 544 322 (1%) | 44 116 848 (22%) | 1 043 942 (1%) | 4 880 809 (2%) | 36 882 851 | 36 523 467 (18%) | 359 384 (0%) | 0.2 |

^a^, Data were obtained from the National Health Data Network (OpenDataSUS), Ministry of Health, Federal Government of Brazil.

The data correspond to the period from January 17, 2021, to June 27, 2024. The complete dataset is available at: <https://www.gov.br/saude/pt-br/>. Data are presented as absolute numbers (N) and percentage (%), enabling both quantitative comparison and proportional interpretation. Vaccination coverage was calculated using the formula (number of administered doses / Indigenous population) × 100.


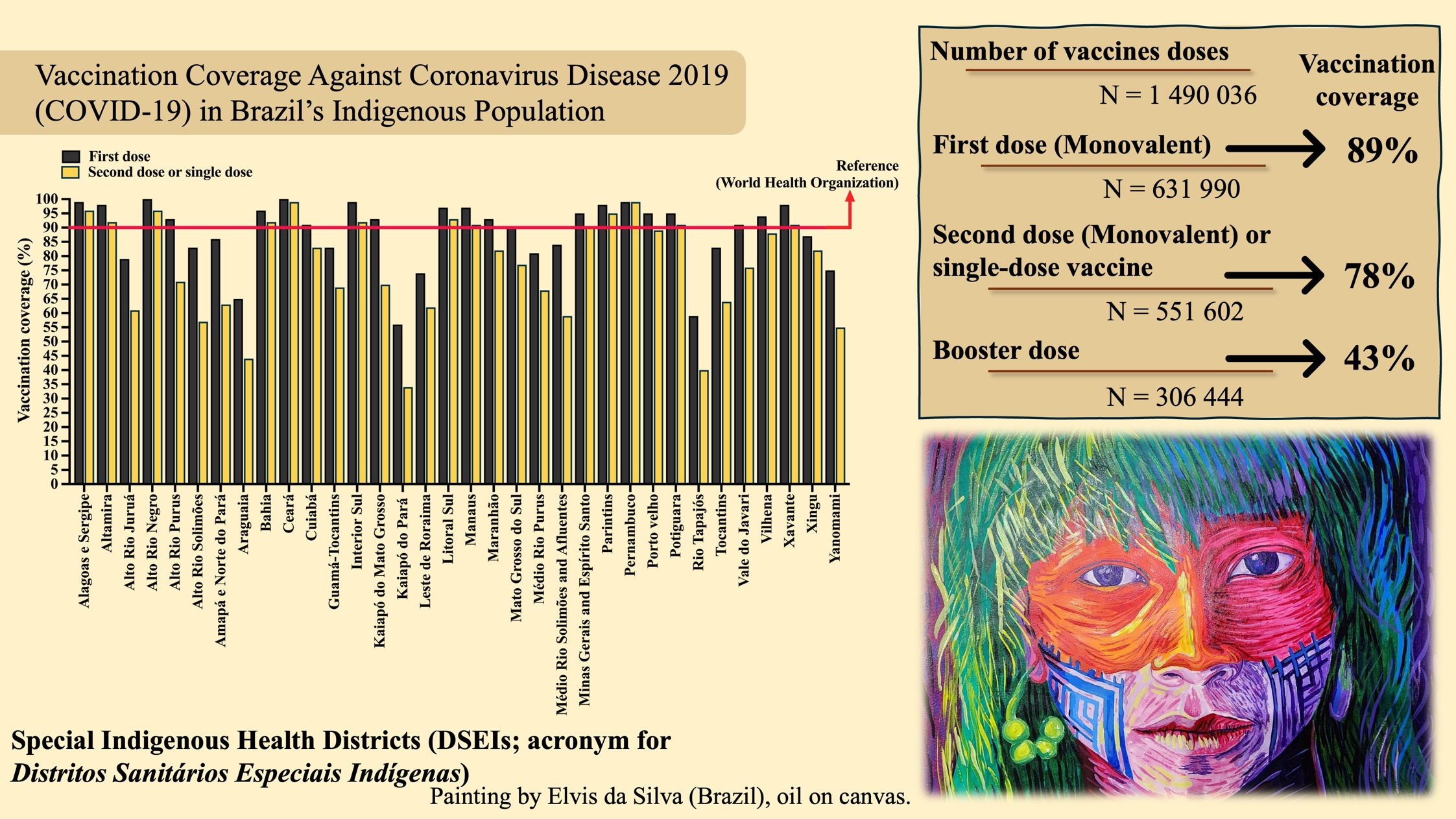


**Graphic Summary**
